# Paspalines C–D and Paxillines B–D: New Indole Diterpenoids from *Penicillium brefeldianum* WZW-F-69

**DOI:** 10.3390/md20110684

**Published:** 2022-10-29

**Authors:** Weiwen Lin, Hanpeng Li, Zhiwen Wu, Jingyi Su, Zehong Zhang, Li Yang, Xianming Deng, Qingyan Xu

**Affiliations:** 1 State Key Laboratory of Cellular Stress Biology, School of Life Sciences, Xiamen University, Xiamen 361102, China; lin_wwen@163.com (W.L.); lihanpeng_bp@163.com (H.L.); wuzhiwen0717@foxmail.com (Z.W.); sjysudi@126.com (J.S.); zhangzh_orange@163.com (Z.Z.); yang_li0712@163.com (L.Y.); xmdeng@xmu.edu.cn (X.D.); 2 State-Province Joint Engineering Laboratory of Targeted Drugs from Natural Products, Xiamen University, Xiamen 361102, China

**Keywords:** marine fungus, *Penicillium brefeldianum*, indole diterpenoids, paspalines, paxillines, cytotoxicity

## Abstract

Five new indole diterpenoids named paspaline C–D (**1**–**2**) and paxilline B–D (**3**–**5**), as well as eleven known analogues (**6**–**16**), were identified from fungus *Penicillium brefeldianum* strain WZW-F-69, which was isolated from an abalone aquaculture base in Fujian province, China. Their structures were elucidated mainly through 1D- and 2D-NMR spectra analysis and ECD comparison. Compound **1** has a 6/5/5/6/6/8 hexacyclic ring system bearing 2,2-dimethyl-1,3-dioxocane, which is rare in natural products. Compound **2** has an unusual open F-ring structure. The cytotoxic activities against 10 cancer cell lines and antimicrobial activities against model bacteria and fungi of all compounds were assayed. No compound showed antimicrobial activity, but at a concentration of 1 μM, compounds **1** and **6** exhibited the highest inhibition rates of 71.2% and 83.4% against JeKo-1 cells and U2OS cells, respectively.

## 1. Introduction

Indole diterpenoids are a family of secondary metabolites featuring a common core structure comprising an indole derived from indole-3-glycerol phosphate, and a cyclic diterpene skeleton derived from geranylgeranyl diphosphate. A select group of ascomycetous fungi, including *aspergillus*, *penicillium*, and zygomycetous fungi, is the main producer [1,2]. They were known initially for tremorgenic and neurotoxic activities partly due to their inhibition of potassium ion channels in the nervous systems [3,4], but in recent years, more diverse bioactivities, such as acyl-CoA:cholesterol acyltransferase (ACAT) inhibitors [5], M phase cell cycle inhibitors [6], and in vitro anti-proliferative, anti-insetan activities [7], etc., were discovered, which indicated their potency as drug lead compounds. Among the known indole diterpenoids, paspaline-type and paxiline-type congeners are the largest classes with the most multitudinous structures [8]. Paspline (**6**) is the simplest member of complex indole diterpenoids, and paxilline is the proposed precursor of terpendoles and other indole ditrpenoids, such as lotirems and janthitrems [9].

**Figure 1:**
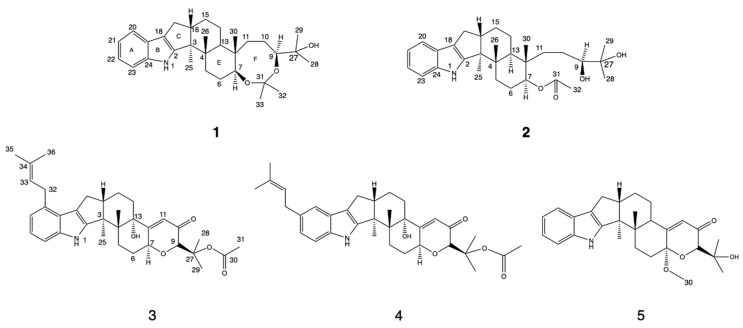
Structures of compounds **1**–**5**.

During our searching for bioactive natural compounds from marine fungi, one fungus, *Penicillium brefeldianum*, strain WZW-F-69, was isolated from an aquaculture base of abalone at Fujian province, China. From the culture, 16 indole diterpenoids, most of which belonged to the paspaline and paxiline types, were identified; thereinto, 5 were elucidated for the first time (Figure 1). Their cytotoxic and antimicrobial activities were tested, some of them showed to be biologically active. Herein, the structural elucidation and bioassay assay are described. 

## 2. Results and Discussion

### 2.1. Structure Elucidation of the New Compounds

The ethyl acetate extract of the fermentation broth of WZW-F-69 was chromatographed on preparative HPLC columns, Sephadex LH-20 to give compounds **1**–**16**.

Paspaline C (**1**) was obtained as a colorless solid. The HR-ESI-MS analysis exhibited [M + H]^+^ ion at *m*/*z* 480.3474 (calculated 480.3472, C_31_H_46_NO_3_^+^, error: 0.2 ppm), which was appropriate for the molecular formula C_31_H_45_NO_3_, indicating 10 degrees of unsaturation. The ^1^H-NMR spectrum (Table 1) revealed the presence of one exchangeable proton (1-NH), four aromatic protons (H-20–H-23), and seven single methyl groups. ^13^C-NMR spectrum showed 31 signals, classified by DEPT and HSQC analysis as nine none-protonated carbons including four olefinic carbons (C-2, C-18, C-19 and C-24), one acetal carbon (C-31), one oxygenated *sp^3^* carbon (C-27), three non-oxygenated *sp^3^* carbons (C-3, C-4 and C-12), and eight methine groups, including four olefinic methines (C-20–C-23) and four *sp^3^* methine carbons (C-7, C-9, C-13 and C-16).

**Figure 2:**
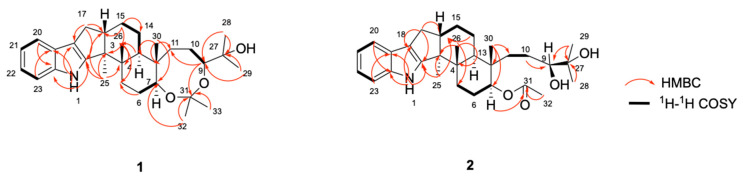
Key HMBC and key COSY correlations of **1** and **2**.

The H-H COSY from H-20 to H-23, as well as the HMBC correlations (Figure 2) from H-1 to C-2/C-18/C-19/C-24, hinted at the presence of a 2,3-disubstituted indole residue (ring A and B). The indole molecule had six degrees of unsaturation; the remaining four degrees of unsaturation indicated that compound **1** has a hexacyclic skeleton. Further analysis of the HMBC correlation from H_3_-25 to C-2/C-3/C-4/C-16 and from H-17 to C-2/C-3/C-16/C-18 revealed that ring C was *ortho*-fused with ring B at C-2 and C-18. Ring D and ring E were established mainly by the COSY correlations from H-13 to H-15 through H-14, and HMBC correlations from H_3_-26 to C-3/C-4/ C-5/C-13, as well as H_3_-30 to C-7/C-11/C-12/C-13. A sub-structure featuring a 6/5/5/6/6-fused system (ring A–E) search resulted in many hits, most of which were indole diterpenoids, mainly the derivatives of paspaline (**6**), which was co-isolated with **1**. Detailed comparison of the NMR data between **1** and **6** showed that compound **1** had an additional 2-oxypropyl unit. The observed HMBC correlations from H-9 to C-31 and from H-32 to C-7 established a 2,2-dimethyl-1,3-dioxocane ring (ring F), so the planar structure of compound **1** was solved. It had a 6/5/5/6/6/8 hexacyclic system. 

**Figure 3:**
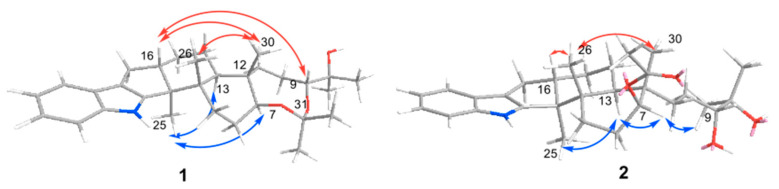
Key NOESY correlations of **1** and **2**.

The relative configuration of **1** was determined mainly by analysis of the NOESY spectrum (Figure 3). The NOE correlations of H-16 and H_3_-26 with H_3_-30 established the *β*-orientations of H-16/H_3_-26/H_3_-30. The correlations of H-7 and H_3_-25 with H-13 indicated the *α*-orientations of H-7/H-13/H_3_-25, which was same as those of known compound **6**. However, the configuration of C-9 in compound **1** needs to be reconfirmed, as the cyclization of ring F might transform it. Careful analysis of the NOE spectrum revealed the correlation of H-13/H_3_-33, H_3_-32/H_3_-28, and H-26/H_3_-29; the three correlations established the *α*-orientation of H-9, then the relative configuration (RC) of compound **1** was 3S*, 4S*, 7S*, 9S*, 12S*, 13R*, and 16S*.

Paspaline D **(2**) had the molecular formula of C_30_H_43_NO_4_ established by the HR-ESI-MS analysis which exhibited the [M + H]^+^ ion at m/z 504.3081 (calculated 504.3084, C_30_H_43_NNaO_4_^+^, error: 0.2 ppm). Comparison of the ^1^H- and ^13^C- NMR spectra of **1** and **2** showed two similar sets of data, the main differences being C-7 and C-31. For compound **1**, C-31 (*δ*106.5 ppm) was an aldehyde acetal carbon, and H-7 was a typical oxygenous chemical shift (*δ*3.59 ppm), while in compound **2**, C-31 (*δ*170.7 ppm) was a carbonyl carbon, and the chemical shift of H-7 (*δ*4.80 ppm) shifted downfield obviously. Detailed analysis of 2D-NMR spectra (Figure 2) revealed that compound **2** had an identical 6/5/5/6/6 system, but had no ring F; an acetoxyl group and a 3, 4-dihydroxy-4-methylpentyl group were attached at C-7 and C-12 with ring E, respectively, so its planar structure was established.

The relative configuration of **2** was established by analysis of the NOE spectrum (Figure 3). The *α* orientations of H-7/H-13/H_3_-25 and *β* orientations of H-16/H_3_-26/H_3_-30 were the same as those in compound **1**. The H-7/H-9 correlation helped in confirming the configuration of C-9 as S*.

**Figure 4:**
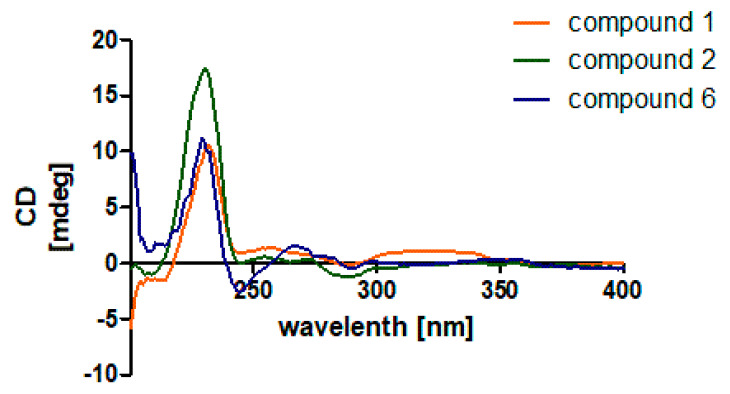
ECD spectra of compound **1**, **2** and **6**.

The ECD experiment was used to determine the absolute configuration (AC) of compounds **1** and **2** (Figure 4). A negative cotton curve at 230–250 nm corresponds to the AC of 3*S*, 4*S*, 13*R* and 16*S* of indole diterpenoids with the 6/5/5/6/6 skeleton of ring A-E as the π→π* transition of the indole ring [10]. Compounds **1**, **2** and **6** had the same negative at about 230 nm (Figure 4) and positive at about 260 nm. Based on the result and biosynthetic considerations, the AC of compounds **1** and **2** was established as 3*S*, 4*S*, 7*S*, 9*S*, 12*S*, 13*R*, and 16*S.* This was confirmed by single crystal X-ray crystallographic analysis of compound **6** (Figure S69).

Paxilline B (**3**) had a molecular formula of C_34_H_43_NO_5_ based on the HR-ESI-MS analysis which exhibited an [M + H]^+^ ion at *m*/*z* 546.3217 (calculated 546.3214, C_34_H_44_NO_5_^+^, error: 0.3 ppm). The ^1^H- and ^13^C-NMR spectra of **3** (Table 2) are very similar to those of compound **7**, O-acetylpaxilline, which revealed that compound **5** is an indole diterpene compound. The ^1^H-NMR spectrum at *δ* 6.88 (dd, *J* = 7.8, 0.8) /7.03 (dd, *J* = 8.1, 7.3)/7.01 (dd, *J* = 8.1, 1.0) suggested a 1, 2, 3-trisubstitued phenyl nucleus, indicating an isopentenyl group located at potion 20 or 23 of indole deterpenoid skeleton. The HMBC correlations of H-21 to C-32, CH_3_-35 and CH_3_-36 to C-33 and C-34 confirmed a 3-methyl-2-butenyl unit at position 20, so the planar structure of **3** was 20-prenylated -27-*O*-acetyl paxilline.

Paxilline C (**4**) had the same molecular formula C_34_H_43_NO_5_ as compound **3** based on (+)-HR-ESI-MS [M + H] ^+^ value 546.3220. The two compounds had highly similar ^1^H-NMR and ^13^C-NMR spectra (Table 2), except for the position of the isopentenyl group. Detailed analysis of 2D-NMR spectra (Table S4) revealed that for compound **4,** the isopentenyl is at C-21, so the planar structure of **4** is 21-prenylated -27-*O*-acetyl paxilline.

The relative configurations of **3** and **4** can be inferred through NOE spectra analysis. Two compounds had the same NOE correlations from H-16 to H_3_-26, from H-7 to H-9, and H_3_-25. Their ECD spectra are highly consistent with those of compound **7** (Figure S63), indicating that compounds **3** and **4** have the absolute configuration of 3*S*, 4*R*, 7*S*, 9*R*, 13*S*, and 16*S*.

Paxilline D (**5**) was obtained as a colorless solid, and its molecular formula was determined as C_28_H_35_NO_4_ based on (+)-HR-ESI-MS data [M + H]^+^ 450.2638 (calculated 450.2639 for C_28_H_36_NO_4_^+^, error: 0.1 ppm). It had highly similar ^1^H-NMR and ^13^C-NMR spectra with 13-desoxypaxilline (**10**) (molecular formula: C_27_H_33_NO_3_, Figures S39 and S40). Detailed analysis revealed that compound **5** had an additional methoxy group (δ_H_ 3.40, δ_C_ 49.1), which was consistent with their molecular formulas. HMBC correlations from H_3_-30 to C-7, as well as the typical aldehyde acetal chemical shift of C-7 (δ_C_ 96.5) established the planar structure of **5** as 7-methoxy-13-desoxypaxilline.

The relative structure of compound **5** was determined by NOE through connection from H-9 to H-13, H_3_-30 and H_3_-25, which indicated the same orientation of these atoms (Table S5). Compound **5** showed a negative cotton effect at 200–250 nm in the ECD spectrum, that corresponded to that of paxilline. Therefore, the absolute configuration of compound **5** was assigned as 3*S*, 4*S*, 7*R*, 9*R*, 12*R*, 16*S*. 

**Figure 5:**
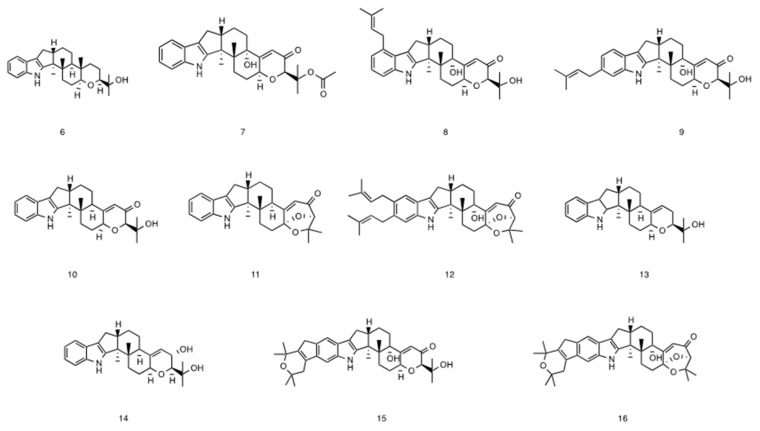
Structures of compounds **6**–**16**.

**Figure 6:**
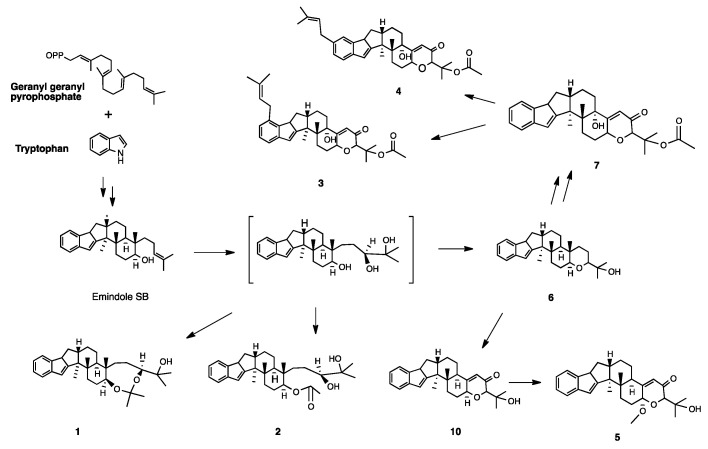
Proposed biosynthesis pathway of compounds **1**–**5**.

In addition to compounds **1**–**5**, 11 congeners—paspaline (**6**) (Figure 5) [11], 1’-*O*-acetyl paxilline (**7**) [12], 20-prenylated paxilline (**8**) [13], 22-prenylated paxilline (**9**) [14], 13-deoxy-paxilline (**10**) [15], paspalicine (**11**) [16], sherinine K (**12**) [17], 3-deoxo- 4b-deoxy paxilline (**13**) [18], 10-hydroxy-13-desoxy paxilline(**14**) [19], pyrapaxilline (**15**) [20], and shearinine F(**16**) [21]—were also isolated and identified. Their structures were determined mainly by analysis of their NMR spectra and comparison with the literature. Based on the substitution around the phenyl ring of the indole moiety and the olefinic bond of ring F, 16 compounds can be divided into 4 groups: compounds **1**, **2** and **6** belong to the paspaline group, which lack substitution on the indole ring and have no olefinic bond on ring F; compounds **5**, **7**, **10**, **11**, **13** and **14** belong to the paxilline group which have an olefinic bond on ring F; compounds **3**, **4**, **8** and **9** are the 3rd group which have one isopentene group on the indole ring; and compounds **12**, **15** and **16** have two isopentyl substituent groups on the indole ring. The biogenetic pathway of the five new compounds is proposed in Figure 6. Emindole SB is likely a biosynthetic precursor of 6 and other advanced indole diterpenoids.

### 2.2. Bioactivities of Compounds

Compounds **1**–**16** were evaluated for their antimicrobial and cytotoxic activities with the cell lines and microbial strains in our lab. All tested compounds showed no activities against *Escherichia coli* CMCC44103, *Staphlococcus aures* CMCC26003, *Aspergillus niger* ACCC3005, and *Candida albicans* AS2.538 by the disk diffusion method at 10 mg/mL, nor cytotoxic activity at 1 μM against MDA-MB-231 (human breast cancer cells), A375 (human melanoma cells), BGC-823 (human pancreatic cancer cells), SKGT4 and Kyle 450 (two human esophagus cancer cell) lines. However, compounds **1** and **6** exhibited moderate inhibitory activity against HepG-2 (human hepatoma carcinoma cells), U2OS (human osteosarcoma cells), MCF-7 (human breast cancer cells), Jeko-1 (human mantle cell lymphoma cells) and HL-60 (human acute promyelocytic leukemia cells). Their average inhibition rates were in the range of 50% to 80% at a concentration of 1 μM (Table 3). Our result is in line with the literature [3,22] that paspline- and paxilline-type indole diterpenoids seldom have anti-microbial activity, while some of them have cytotoxic activity, which differs from different cancer cell lines. 

## 3. Materials and Methods

### 3.1. General Apparatus

The NMR data were recorded on Bruker Advance-III spectrometer (Bruker, Karlsruhe, Germany). Mass spectra were performed on the Waters 2767 LC-MS system using the Nova-pak silica column (Waters, Milford, CT, USA). HR-ESI-MS was recorded on Thermo Scientific Q Exactive HF Orbitrap-FTMS (Thermo Fisher, Waltham, MA, USA). Analytic HPLC was operated on Agilent 1200 series liquid chromatography with an Agilent XDB-C18 column (9.4 × 250 mm, 5 μm) and UV detector (Agilent, Santa Clara, CA, USA). Semi-preparative HPLC was operated on Welch Sail 1000 liquid chromatography with Prep RP-18 column (28 × 4 cm) and UV detector (Welch, Baltimore, MD, USA). Mid-press reverse phase chromatography was operated on Buchi Switzerland system with BUCHI RP-18 column (20 × 4 cm, 170 g) (Büchi, Uster, Switzerland). Column chromatography (CC) was performed with silica gel (300–400 mesh, Yantai Jiangyou silicon development Co., Ltd., Yantai, China) and Sephadex LH-20 (GE Healthcare, Chicago, IL, USA).

### 3.2. Fungal Material

The fungal strain WZW-F-69 was isolated from soil near an abalone aquaculture base of Fujian province, China (N 23°47′727″, E 117°29′079″), in August 2015. After being purified, the strain was identified with the ITS gene sequence (Sequence S1) to be *Penicillium brefeldianum* (100% consistent with Genebank KM243921.1). The strain was preserved in our lab at −80 °C. 

### 3.3. Fermentation and Extraction

For chemical investigation, the fungal strain WZW-F-69 was cultivated in 100 mL liquid seawater potato-dextrose medium (including extract of potato 200 g, glucose 20 g, sea salt 30 g, water 1 L), 220 rpm, at 28 °C for 5–7 days to prepare the seed culture. Then, 5 mL of the seed solution was inoculated on solid rice culture media (150 g rice in 150 mL water) in a 2.5 L stainless steel box. A total of 3 kg of the rice media was incubated at 28 °C for 20 days. The rice culture media were collected and soaked in ethyl acetate (EA) overnight. After the solvent was removed in vacuum, it yielded a crude extract (20 g). The crude extract was redissolved in MeOH: petroleum ether (PE, 1:1, *v*/*v*) three times. The combined MeOH extract (10 g) was grouped by the RP-18 silica gel column mid pressure chromatography (28 × 4 cm, 170 g), and the elute solution changed from 100% H_2_O to 100% MeOH in 2 h, 30 mL/min. 

### 3.4. Isolation and Purification

The 70% MeOH elution, component D (802.8 mg), was purified by Sephadex LH-20 (2.5 × 140 cm, 140g in MeOH, 15 s/drip, 1 tube/h) and gave 7 fractions (D-1 to D-7). Compound **2** (3.2 mg) was purified from D-2 with Agilent HPLC (MeOH 80%, 5 µm, 4.6 × 250 mm, 3 mL/min). D-3 afforded 11 fractions (D-3-1 to D-3-11) with Sephadex LH-20 (2 × 80 cm, 80 g, in acetone, 15 s/drip, 1 tube/h). Compounds **1** (1.0 mg), **6** (9.6 mg) and **14** (3.2 mg) were purified from D-3-4, D-3-9 and D-3-6, respectively. D-4 afforded 7 fractions (D-4-1 to D-4-7) by Sephadex LH-20 in acetone (2.5 × 15 cm, 140 g, 15 s/drip, 1 tube/h). D-4-1 yielded **7** (58.0 mg), **10** (4.6 mg), **11** (6.3 mg) after being subjected to a silica gel column chromatography (2 × 8 cm, 10 g) with elution of PE:EtoAc (from 35:1 to 0:1 in five hours). Compound **5** (7.3 mg) was yielded from D-4-2 through Agilent HPLC (MeCN/H_2_O, 80%).

The 80% MeOH elution, component E (3.280 g), was purified by Sephadex LH-20 (2.5 × 140 cm, 140 g, in CHCl_2_/MeOH, 1:2, *v*:*v*) then by Sephadex LH-20 (140 g, acetone) and silica gel column chromatography (24 g, PE: EtoAc, 100:0 to 0:100 in 3 h), afforded **3** (3.2 mg) and **4** (1.0 mg).

The 90% MeOH eluted part, component F (1.075 g), yielded 11 fractions (F-1 to F-11) after Sephadex LH-20 chromatography (140 g, in MeOH). F-8 was subjected to silica gel CC (4 g, PE:EtoAc, 100:0 to 0:100 in two hours) to gain 3 sub-fractions (F-8-1 to F-8-3). F-8-2 afforded **12** (2.1 mg) by Agilent HPLC (MeCN-H_2_O, 90%, 5 µm, 4.6 × 250 mm, 3 mL/min). 

The potion of 90–100% MeOH, component G (1.662 g), gave **9** (5.3 mg), **13** (1.8 mg), **15** (4.1 mg) and **16** (5.0 mg) after Sephadex LH-20 (2.5 × 140 cm, 140 g, in MeOH and 2.5 × 140 cm, 140 g, in acetone).

Another patch of the MeOH extract named component H (1.5 g) followed Sephadex LH-20 (2.5 × 140 cm, 140g, in MeOH), silica gel CC (30 g, PE: EtoAc, 100:0 to 0:100 in 3 h) and Agilent HPLC (MeCN/H_2_O, 80%) and yielded **8** (1.0 mg). 

Paspaline C (**1**): colorless solid, [α]_25_^D^: 0 (*c* 1.0, MeOH), HR-ESI-MS at *m*/*z* 480.3474 [M + H]^+^, calculated for C_31_H_45_NO_3_, 480.3472. UV (MeOH), λ_max_ (log *ε*): 284 (1.4), 225 (5.1). IR (KBr) ν_max_ 3430, 2939, 2253, 1651, 1455, 1086, 1049, 1025, 1004, 824, 762, 615. For ^1^H-NMR(CDCl_3_, 600 MHz) and ^13^C-NMR(CDCl_3_, 150 MHz) data, see Table 1; for UV spectrum, see Figure S64. 

Paspaline D (**2**): colorless solid, [α]_25_^D^: +34 (*c* 1.0, MeOH), HR-ESI-MS at *m*/*z* 504.3081 [M + Na]^+^, calculated for C_35_H_45_NO_4_Na, 504.3084. UV (MeOH), λ_max_ (log *ε*): 280 (1.2). IR (KBr) ν_max_ 3440, 2974, 2114, 1634, 1456, 1364, 1276, 1160, 1048, 1032, 911, 471. For ^1^H-NMR (CDCl_3_, 600 MHz) and ^13^C-NMR (CDCl_3_, 150 MHz) data, see Table 1; for UV spectrum, see Figure S65. 

Paxilline B (**3**): colorless solid, [α]_25_^D^: +24 (*c* 1.0, MeOH), HR-ESI-MS at *m*/*z* 546.3217 [M + H]^+^, calculated for C_34_H_44_NO_5_, 546.3214. UV. UV (MeOH), λ_max_ (log *ε*): 285 (1.0), 225 (3.8). IR (KBr) ν_max_ 3430, 2948, 2843, 1658, 1453, 1377, 1179, 1113, 1020, 635. ^1^H-NMR (CDCl_3_, 600 MHz) and ^13^C-NMR (CDCl_3_, 150 MHz) data, see Table 2; for UV spectrum, see Figure S66. 

Paxilline C (**4**): colorless solid, [α]_25_^D^: +60 (*c* 1.0, MeOH), HR-ESI-MS at *m*/*z* 546.3220 [M + H]^+^, calculated for C_34_H_44_NO_5_, 546.3214. UV (MeOH), λ_max_ (log *ε*): 290 (1.1), 240 (4.3). IR (KBr) ν_max_ 3403, 2982, 2937, 2849, 1709, 1676, 1624, 1449, 1368, 1259, 1124, 1025, 960, 928, 742, 709, 606, 583. For ^1^H-NMR (CDCl_3_, 600 MHz) and ^13^C-NMR (CDCl_3_, 150 MHz) data, see Table 2; for UV spectrum, see Figure S67. 

Paxilline D (**5**): colorless solid, [α]_25_^D^: 0 (*c* 1.0, MeOH), HR-ESI-MS at *m*/*z* 450.2638 [M + H]^+^, calculated for C_28_H_36_NO_4_, 450.2639. UV (MeOH), λ_max_ (log *ε*): 230 (1.5). IR (KBr) ν_max_ 3400, 2928, 1658, 1448, 1373, 1303, 1258, 1127, 1032, 742. For ^1^H-NMR (CDCl_3_, 600 MHz) and ^13^C-NMR (CDCL_3_, 150 MHz) data, see Table 2; for UV spectrum, see Figure S68. 

### 3.5. Antimicrobial Activity

The antimicrobial activity was evaluated with the disk diffusion methodology [23]. Four indicator organisms (*Staphococcus aureus* CMCC26003, *Escherichia coli* CMCC44103, *Aspergillus niger* ACCC3005, *Canidida albicans* AS2.538) were used. The spores at 1×10^6^/mL were poured onto culture media on a Petri dish (φ = 9 cm): PDA medium for fungal indicators, and Luria–Bertain (LB) medium for bacterial indicators. The sterilized filter paper (φ = 5 cm) with 5 µL of compound solution at 10 mg/mL was put on the surface of the culture medium; 24–48 h later, the inhibition diameters were measured. Amphotricin B and gentamicin at 1 mg/mL were used as positive controls for the fungal and bacterial indicators, respectively. 

### 3.6. Cytotoxic Activity

A375, MDA-MB-231, HepG2, BGC823, U2OS and MCF-7 cell lines were grown in DMEM medium. SKGT4, Kyse450, JeKo-1 and HL60 cells were grown in RPMI1640 medium. Two media were supplemented with 10% FBS and 1% penicillin or streptomycin. Then, 198 µL of the medium with about 5000 cancer cells was plated in each well of a 96-well plate. Then, 2 µL of the test compound at 10 mM in DMSO was added in triplicate. Forty-eight hours later, the cell viability was determined using CellTiter 96^®^ AQ_ueous_ Assay Reagents according to the instruction manual at 490 nm. Data were normalized to the control group (DMSO).

## 4. Conclusions

In summary, 5 new indole diterpenoids (**1**–**5**) and 11 known congeners (**6**–1**6**) were identified from sea-mud-derived fungal *Penicillium Brefeldianum* WZW-F-69. Even though the main structural elements resemble the reported indole diterpenes, paspaline C (**1**) has a rare 2,2-dimethyl-1,3-dioxocane ring F, while paspaline D (**2**) has an unusual open ring F structure. Paxilline B (**3**) and paxilline C (**4**) are isopentenyl ring A derivatives of paxilline, while paxilline D (**5**) has an aldehyde acetyl carbon (C-7), which makes them chemically unique. Although the AC of new compounds was established mainly by the comparison of ECD spectra with known compounds **6** and **7**, the consistence with known compounds, such as 6-hydroxylpaspslinine [3], 4a-demethylpaspaline-4a-carboxylic acid [7], whose AC was confirmed with experimental and calculated ECD, supported our assignment. Compounds **1** and **6** exhibited cytotoxic activity against several cancer cell lines. In summary, the identification of diverse structural compounds from WZW-F-69 illustrates the huge potential of finding more new bioactive lead compounds from marine fungi for future research.

## Figures and Tables

**Table 1:** ^1^H (600 MHz) and ^13^C NMR (150 MHz) data of compounds **1** and **2** (CDCl_3_, *δ* in ppm, *J* in Hz).

No.	1		2	
	^1^H	^13^C	^1^H	^13^C
1	7.74, s		7.77, s	
2		150.8, C		150.6, C
3		53.1, C		53.1, C
4		39.3, C		39.3, C
5	1.94, dd (11.3, 5.8) 1.62, m	33.6, CH_2_	2.05, dd (13.7,4.6) 2.01, dd (12.5, 5.4)	33.1, CH_2_
6	1.89, dd (14.7, 2.7) 1.87, m	26.9, CH_2_	1.90, m	23.8, CH_2_
7	3.59, dd (9.8, 5.9)	72.8, CH	4.80, dd (10.2, 5.9)	75.5, CH
9	3.68, dd (8.3, 4.6)	84.1, CH	3.57, dd (8.5, 4.3)	83.6, CH
10	1.34, dd (12.1, 5.6) 1.32, m	22.2, CH_2_	1.59, m	22.6, CH_2_
11	1.71, td (12.9, 3.0) 1.41, m	33.9, CH_2_	1.55, m 1.32, m	34.4, CH_2_
12		39.3, C		40.2, C
13	1.69, s	41.4, CH	1.78, dd (12.7, 3.1)	40.4, CH
14	1.81, d (12.6) 1.64, brs	25.2, CH_2_	1.82, m 1.65, td (12.9, 4.1)	25.1, CH_2_
15	1.71, m 1.49, td (12.4, 4.4)	23.0, CH_2_	1.59, m	22.6, CH_2_
16	2.78, m	47.8, CH	2.78, m	48.7, CH
17	2.70, dd (13.2, 6.5) 2.35, dd (13.2, 10.6)	27.5, CH_2_	2.70, dd (13.2, 6.4) 2.36, dd (13.2, 10.6)	27.5, CH_2_
18		118.4, C		118.3, C
19		125.4, C		125.1, C
20	7.45, ddd (6.6, 3.2, 1.0)	118.3, CH	7.44, m	118.4, CH
21	7.09, m	119.6, CH	7.09, m	119.6, CH
22	7.10, m	120.5, CH	7.09, m	120.5, CH
23	7.32, ddd (6.4, 2.5, 0.8)	111.4, CH	7.32, m	111.5, CH

**Table 2:** ^1^H (600 MHz) and ^13^C NMR (150 MHz) data of compounds **3**–**5** (CDCl_3_, *δ* in ppm, *J* in Hz).

No.	3		4		5	
	^1^H	^13^C	^1^H	^13^C	^1^H	^13^C
1	7.78, s		7.70, s		7.79, s	
2		151.0, C		151.9, C		149.3, C
3		50.5, C		50.7, C		50.5, C
4		43.1, C		43.1, C		42.4, C
5	2.80, td (13.5, 4.9) 1.49, m	28.1, CH	2.78, td (13.7, 4.4) 1.47, m	28.0, CH	2.15, td (13.7, 6.4) 1.58, m	31.4, CH
6	2.31, m 1.95, m	28.3, CH_2_	2.31, m 1.93, m	28.3, CH_2_	2.44, m 1.90, dd (14.6, 4.3)	28.8, CH_2_
7	4.84, m	72.9, CH	4.84, m	72.9, CH		96.5, C
9	4.85, brs	80.4, CH	4.85, brs	80.4, CH	4.06, s	76.9, CH
10		195.3, C		195.3, C		198.5, C
11	5.85, d (2.1)	120.1, CH	5.84, d (2.0)	120.1, CH	5.81, d (2.0)	122.1, CH
12		166.1, C		166.1, C		165.1, C
13		77.5, C		77.5, C	2.83, m	42.0, CH
14	2.08, m 1.66, m	34.4, CH_2_	2.07, m 1.67, m	34.4, CH_2_	1.72, m 1.52, m	25.5, CH_2_
15	2.07, m	20.9, CH_2_	2.05, m 1.80, m	20.9, CH_2_	1.86, m	24.1, CH_2_
16	2.89, d (2.8)	49.6, CH	2.85, m	49.6, CH	2.85, m	49.0, CH
17	2.91, d (6.2) 2.62, m	29.0, CH_2_	2.74, dd (13.0, 6.3) 2.45, dd (13.2, 10.9)	27.2, CH_2_	2.74, dd (13.4, 6.4) 2.45, m	27.3, CH_2_
18		116.9, C		117.1, C,		118.4, C
19		124.4, C		125.6, C		125.0, C
20		133.1, C	7.25 (brs)	117.6, CH	7.47, dd (6.8, 2.2)	118.5, CH
21	6.88, dt (7.3, 0.8)	119.0, CH		133.3, C	7.12, m	120.8, CH
22	7.03, dd (8.1, 7.3)	121.0, CH	6.94, dd (8.2, 1.7)	121.4, CH	7.12, m	119.8, CH
23	7.17, dd (8.1, 1.0)	109.3, CH	7.23, dd (8.1, 1.0)	111.3, CH	7.33, dd (6.8, 2.2)	111.4, CH
24		139.7, C		138.2, C		140.0, C
25	1.34, s	16.2, CH_3_	1.33, s	16.2, CH_3_	1.12, s	14.7, CH_3_
26	1.08, s	19.8, CH_3_	1.05, s	19.7, CH_3_	1.03, s	15.9, CH_3_
27		81.9, C		81.9, C		72.4, C
28	1.46, s	22.8, CH_3_	1.46, s	22.8, CH_3_	1.29, s	24.0, CH_3_
29	1.68, s	23.8, CH_3_	1.68, s	23.8, CH_3_	1.35, s	26.7, CH_3_
30		170.8, C		170.8, C	3.40, s	49.1, CH_3_
31	2.06, s	22.3, CH_3_	2.06, s	22.3, CH_3_		
32	3.64, d (7.3)	32.0, CH_2_	3.43, d (7.3)	34.5, CH_2_		
33	5.43, m	123.7, CH	5.40, m	124.6, CH		
34		131.8, C		131.4, C		
35	1.76, s	25.8, CH_3_	1.76, s	25.8, CH_3_		
36	1.77, s	17.8, CH_3_	1.77, s	17.8, CH_3_		

**Table 3:** The average inhibition rate of compounds **1** and **6** against cell lines (%).

	HepG-2	U2OS	MCF 7	JeKo-1	HL-60
Compound **1**	55.1	56.1	56.4	71.2	65.8
Compound **6**	52.4	83.4	47.5	72.4	60.3

## Supplementary Materials

The following are available online at https://www.mdpi.com/1660-3397/20/11/684/s1, Sequence S1: Taxonomy of *Penicillium Brefeldianum* WZW-F-69, Tables S1–S5: NMR (600 MHz, CDCl3) data for Compounds **1**–**5**, Figures S1–S30: 1D and 2D NMR spectra of compounds **1**–**5**, Figures S31–S52: ^1^H-NMR, ^13^C-NMR of compounds **6**–**16**, Figures S53–S57: HR-ESI-MS reports of **1**–**5**, Figures S58–S62: ECD spectra of compounds **1**–**5**, Figure S63: ECD spectrum of compounds **3**, **4** and **7**, Figures S64–S68: UV spectra of compounds **1**–**5**, Figure S69: x-ray single crystal diffraction structure of compound **6**.
