# Supplementary material for: Paspalines C–D and Paxillines B–D: New Indole Diterpenoids from Penicillium brefeldianum WZW-F-69
Source: Mar Drugs. 2022 Oct 29;20(11):684. doi: 10.3390/md20110684 (PMC9697303; doi:10.3390/md20110684)
Supplement: Supplementary file 1 [file marinedrugs-20-00684-s001.zip › marinedrugs-1973261-supplementary.pdf]

Supplementary Material

Paspalines C–D and Paxillines B–D: New Indole Diterpenoids from *Penicillium brefeldianum* WZW-F-69

Weiwen Lin [1,2], Hanpeng Li [1,2], Zhiwen Wu [1,2], Jingyi Su [1,2], Zehong Zhang [1], Li Yang [1], Xianming Deng [1,2] and Qingyan Xu [1,2,*]

[1] State Key Laboratory of Cellular Stress Biology, School of Life Sciences, Xiamen University, Xiamen 361102, China
[2] State-Province Joint Engineering Laboratory of Targeted Drugs from Natural Products, Xiamen University, Xiamen 361102, China
* Correspondence: xuqingyan@xmu.edu.cn; Tel.: +86-592-2180328

Table of contents

1. Taxonomy of *Penicillium brefeldianum* WZW-F-69 ……………………………6

2. Tabulated NMR data ………………………………………………………..7

3. NMR spectra ………………………………………………………………17

4. HR-ESI-MS data ……………………………………………………...43

5. ECD spectra……………………………………………………………………48

6. UV spectra ……………………………………………………………… 52

7. Strucutre of X-ray single crystal diffraction……………………………………...54

List of Sequence

Sequence S1……………………………………………………………………..6

List of Tables

Table S1. NMR (600 MHz, $CDCl_3$) data for paspaline C (**1**) ………………………..7

Table S2. NMR (600 MHz, $CDCl_3$) data for paspaline D (**2**) ……………………… 9

Table S3. NMR (600 MHz, $CDCl_3$) data for paxilline B (**3**) ………………………11

Table S4. NMR (600 MHz, $CDCl_3$) data for paxilline C (**4**) ………………………..13

Table S5. NMR (600 MHz, $CDCl_3$) data for paxilline D (**5**) ………………………..15

List of Figures

Figure S1. ^{1}H NMR (600 MHz, $CDCl_3$) spectrum of paspaline C (**1**) ……………...17

Figure S2. ^{13}C NMR (150 MHz, $CDCl_3$) spectrum of paspaline C (**1**) ……………...17

Figure S3. HSQC spectrum of paspaline C (**1**) ……………………………………..18

Figure S4. HMBC spectrum of paspaline C (**1**) ……………………………………18

Figure S5. ^{1}H -^{1}H COSY spectrum of paspaline C (**1**) ……………………………19

Figure S6. NOE spectrum of paspaline C (**1**) ………………………………………19

Figure S7. ^{1}H NMR (600 MHz, $CDCl_3$) spectrum of paspaline D (**2**) ……………...20

Figure S8. ^{13}C NMR (150 MHz, $CDCl_3$) spectrum of paspaline D (**2**) ……………20

Figure S9. HSQC spectrum of paspaline D (**2**) ……………………………………21

Figure S10. HMBC spectrum of paspaline D (**2**) ………………………………… 21

Figure S11. ^{1}H -^{1}H COSY spectrum of paspaline D (**2**) …………………………..22

Figure S12. NOE spectrum of paspaline D (**2**) ……………………………………22

Figure S13. ^{1}H NMR (600 MHz, $CDCl_3$) spectrum of paxilline B (**3**) …………… 23

Figure S14. ^{13}C NMR (150 MHz, $CDCl_3$) spectrum of paxilline B (**3**) ……………23

Figure S15. HSQC spectrum of paxilline B (**3**) ……………………………………24

Figure S16. HMBC spectrum of paxilline B (**3**)……………………………………24

Figure S17. ^{1}H -^{1}H COSY spectrum of paxilline B (**3**)……………………………25

Figure S18. NOE spectrum of paxilline B (**3**) …………………………………… 25

Figure S19. ^{1}H NMR (600 MHz, $CDCl_3$) spectrum of paxilline C (**4**) ……………26

Figure S20. ^{13}C NMR (150 MHz, $CDCl_3$) spectrum of paxilline C (**4**) …………...26

Figure S21. HSQC spectrum of paxilline C (**4**) ………………………………………27
Figure S22. HMBC spectrum of paxilline C (**4**)…………………………………………27
Figure S23. ^{1}H -^{1}H COSY spectrum of paxilline C (**4**)……………………………28
Figure S24. NOE spectrum of paxilline C (**4**) ………………………………………..28
Figure S25. ^{1}H NMR (600 MHz, $CDCl_3$) spectrum of paxilline D (**5**) ……………29
Figure S26. ^{13}C NMR (150 MHz, $CDCl_3$) spectrum of paxilline D (**5**) …………...29
Figure S27. HSQC spectrum of paxilline D (**5**) ……………………………………...30
Figure S28. HMBC spectrum of paxilline D (**5**)……………………………………...30
Figure S29. ^{1}H -^{1}H COSY spectrum of paxilline D (**5**)…………………………...31
Figure S30. NOE spectrum of paxilline D (**5**) ………………………………………..31
Figure S31. ^{1}H NMR (600 MHz, $CDCl_3$) spectrum of paspaline (**6**)……………..32
Figure S32. ^{13}C NMR (150 MHz, $CDCl_3$) spectrum of paspaline (**6**) ……………32
Figure S33. ^{1}H NMR (600 MHz, $CDCl_3$) spectrum of 1′-O-acetyl paxilline (**7**)…………………………………………………………………..33
Figure S34. ^{13}C NMR (150 MHz, $CDCl_3$) spectrum of 1′-O-acetyl paxilline (**7**) .33
Figure S35. ^{1}H NMR (600 MHz, $CDCl_3$) spectrum of 20-prenylated paxilline (**8**) …………………………………………………………………34
Figure S36. ^{13}C NMR (150 MHz, $CDCl_3$) spectrum of 20-prenylated paxilline (**8**) …………………………………………………………………34
Figure S37. ^{1}H NMR (600 MHz, $CDCl_3$) spectrum of 22-prenylated paxilline (**9**) …………………………………………………………………35
Figure S38. ^{13}C NMR (150 MHz, $CDCl_3$) spectrum of 22-prenylated paxilline (**9**) …………………………………………………………………35
Figure S39. ^{1}H NMR (600 MHz, $CDCl_3$) spectrum of 13-deoxy-paxilline (**10**)..36
Figure S40. ^{13}C NMR (150 MHz, $CDCl_3$) spectrum of 13-deoxy-paxilline (**10**) .36
Figure S41. ^{1}H NMR (600 MHz, $CDCl_3$) spectrum of paspalicine (**11**)…………37
Figure S42. ^{13}C NMR (150 MHz, $CDCl_3$) spectrum of paspalicine (**11**) ………..37
Figure S43. ^{1}H NMR (600 MHz, $CDCl_3$) spectrum of sherinine K (**12**)…………38
Figure S44. ^{13}C NMR (150 MHz, $CDCl_3$) spectrum of sherinine K (**12**) ………..38
Figure S45. ^{1}H NMR (600 MHz, $CDCl_3$) spectrum of 3-deoxo- 4b-deoxy paxillin (**13**) ………………………………………………………………...39

Figure S46. ^{13}C NMR (150 MHz, $CDCl_3$) spectrum of 3-deoxo- 4b-deoxy paxilline (**13**…………………………………………………………….39
Figure S47. ^{1}H NMR (600 MHz, $CDCl_3$) spectrum of 10-hydroxy-13-desoxy paxilline (**14**)…………………………………………………….40
Figure S48. ^{13}C NMR (150 MHz, $CDCl_3$) spectrum of 10-hydroxy-13-desoxy paxilline (**14**) ……………………………………………………40
Figure S49. ^{1}H NMR (600 MHz, $CDCl_3$) spectrum of pyrapaxilline (**15**)………..41
Figure S50. ^{13}C NMR (150 MHz, $CDCl_3$) spectrum of pyrapaxilline (**15**) ………41
Figure S51. ^{1}H NMR (600 MHz, $CDCl_3$) spectrum of shearinine (**16**)…………...42
Figure S52. ^{13}C NMR (150 MHz, $CDCl_3$) spectrum of shearinine (**16**) …………..42
Figure S53. HR-ESI-MS data report of paspaline C (**1**) ……………………………43
Figure S54. HR-ESI-MS data report of paspaline D (**2**) ……………………………44
Figure S55. HR-ESI-MS data report of paxilline B (**3**) …………………………...45
Figure S56. HR-ESI-MS data report of paxilline C (**4**) …………………………...46
Figure S57. HR-ESI-MS data report of paxilline D (**5**) ……………………………47
Figure S58. ECD spectrum of paspaline C (**1**) ……………………………………...48
Figure S59. ECD spectrum of paspaline D (**2**) ……………………………………...48
Figure S60. ECD spectrum of paxilline B (**3**) ………………………………………49
Figure S61. ECD spectrum of paxilline C (**4**) ………………………………………49
Figure S62. ECD spectrum of paxilline d (**5**) ……………………………………….50
Figure S63. ECD spectrum of compound **3**, **4** and **7** ………………………………..51
Figure S64. UV spectrum of paspaline C (**1**) ………………………………………..52
Figure S65. UV spectrum of paspaline D (**2**) ………………………………………..52
Figure S66. UV spectrum of paxilline B (**3**) ………………………………………53
Figure S67. UV spectrum of paxilline C (**4**) ………………………………………...53
Figure S68. UV spectrum of paxilline D (**5**) ………………………………………..54
Figure S69. X-ray single crystal diffracion structure of paspaline (**6**) ……………...54

Sequence S1: Taxonomy of *Penicillium brefeldianum* WZW-F-69

ATGCCCCGGCAACAGCATGATCCGAGGTCAGAGTGTAAAAAATGTACT
TTTTGGACGTCGTCGTTATGAGTGCAAAGCGCGAGATGTACTGCGCTC
CGAAATCAATACGCCGGCTGCCAATTGTTTTGAGGCGAGTCTGCGCGC
AGAGGCGAGACAAACACCCAACACCAAGCAAAGCTTGAAGGTACAAA
TGACGCTCGAACAGGCATGCCCCATGGAATACCAAGGGGCGCAATGT
GCGTTCAAAGATTCGATGATTCACTGAATTCTGCAATTCACACTACTTA
TCGCATTTCGCTGCGTTCTTCATCGATGCCAGAACCAAGAGATCCGTTG
TTGAAAGTTGTAACTATTAAAGTTTTTCAGACGCTGATTGCAACTACA
AAGGGTTTAAATGTGTCCAATCGGCGGGCGGACCCGCCGAGGAAACG
AAGGTACTCAAAAGACATGGGTAAGAGATAGCAGGCAAAGCCTACAA
CTCTAGGTAATGATCCTTCCGCAGGTTCACCCTACGGAAA

Table S1. NMR (600 MHz, $CDCl_3$) data for paspaline C (**1**)

No.	δ_H *(mult., J [Hz], int.)*	δ_c	*HMBC*	*^1H-^1H COSY*	*NOE*
1	7.74 (s)	/	C-2, C-19, C-24	/	H-23
2	/	150.8s	/	/	/
3	/	53.1s	/	/	/
4	/	39.3s	/	/	/
5	1.94 (dd, 11.3, 5.8)	33.6t	C-6	H-13	/
	1.62 (m)		/	/	/
6	1.89 (dd, 14.7, 2.7)	26.9t	C-5, C-7	H-7	/
	1.87 (m)		C-7	H-7	/
7	3.59 (dd, 9.8, 5.9)	72.8d	C-11, C-30	H-6, H-14	H-13
9	3.68 (dd, 8.3, 4.6)	84.1d	C-11, C-29, C-31 (w)	H_3-29	/
10	1.34 (dd, 12.1, 5.6)	22.2t	/	H-9	/
	1.32 (m)				
11	α 1.70(td, 12.9, 3.0)	33.9t	C-4, C-10	/	/
	β 1.41 (m)		/	H-11α	/
12	/	39.9s	/	/	/
13	1.69 (s)	41.4d	C-30	H_3-33	H_3-25
14	1.81 (d, 12.6)	25.2t	C-12, C-26	/	/
	1.64 (bro)		/	H-13	/
15	1.71 (m)	23.0t	/	H-13	/
	1.49 (dd, 12.4, 4.4)				
16	2.78 (m)	48.7d	/	H-17	H_3-26
17	2.70 (dd, 13.2, 6.5)	27.5t	C-2, C-3, C-16, C-18		/
	2.35 (dd, 13.2, 10.6)		C-2, C-18		/
18	/	118.4s	/	/	/
19	/	125.4s	/	/	/
20	7.45 (m)	118.3d	C-22, C-24	H-21	/

21	7.10 (m)	119.6d	C-18, C-19, C-24	H-23, H-20	/
22	7.10 (m)	120.5d	/	/	/
23	7.32 (m)	111.4d	C-19, C-21	H-22	/
24	/	140.0s	/	/	/
25	1.04 (s)	14.7q	C-2, C-3, C-4, C-16	/	H-13
26	1,14 (s)	19.2q	C-3, C-4, C-5	H_2-5	/
27	/	80.1s	/	/	/
28	1.16 (s)	22.9q	C-9, C-27, C-29	H_3-29	/
29	1.30 (s)	26.3q	C-9, C-27, C-28	H_3-28	/
30	0.89 (s)	16.5q	C-7, C-11, C-12, C-13	/	/
31	/	106.5s	/	/	/
32	1.37 (s)	27.1q	C-7 (w), C-31, C-33	/	/
33	1.45 (s)	28.6q	C-31, C-32	/	

Table S2. NMR (600 MHz, $CDCl_3$) data for paspaline D (**2**)

No	δ_H *(mult., J [Hz], int.)*	δ_c	*HMBC*	*^1H-^1H COSY*	*NOE*
1	7.77 (s)	/	C-2, C-16, C-18, C-19	/	H_3-25, H-23
2	/	150.6s	/	/	/
3	/	53.1s	/	/	/
4	/	39.3s	/	/	/
5	2.05 (dd, 13.7, 4.6)	33.1t	C-6, C-26	H_2-6	/
	2.01 (dd, 12.5, 5.4)		/	H-15	H-7
6	1.90 (2H, m)	23.8t	C-5	H-5, H-7	/
7	4.80 (dd, 10.2, 5.9)	75.5d	C-6, C-7, C-11, C-30, C-31	H_2-6	H-13
9	3.57 (dd, 8.5, 4.3)	83.6d	C-11, C-28, C-29	H_3-29	/
10	1.59 (m)	22.6t	C-7	/	/
11	1.55 (m)	34.4t	C-9	/	H-7
	1.32 (m)		/	/	/
12	/	40.2s	/	/	/
13	1.78 (dd, 12.7, 3.1)	40.4d	C-7 (w)	/	H_3-25
14	1.82 (m)	25.1t	C-4 (w)	/	/
	1.65 (td, 12.9, 4.1)		C-16 (w)	/	/
15	1.60 (m, 2H)	22.7t	C-13, C-16	H-16	H-1
16	2.78 (m)	48.7d	C-17	H-14, H-15	H_3-26
17	2.70 (dd, 13.2, 6.4)	27.5t	C-2, C-16, C-18	/	/
	2.36(dd, 13.3,10.6)			/	/
18	/	118.3s	/	/	/
19	/	125.1s	/	/	/
20	7.44 (m)	118.4d	C-22, C-24	H-21	/
21	7.09 (m)	119.6d	C-19, C-23	H-20, H-23	/
22	7.09 (m)	120.5d	C-20, C-24	/	/

23	7.32 (m)	111.5d	C-19, C-21	H-21	H-1
24	/	139.9s	/	/	/
25	1.05 (s, 3H)	14.7q	C-2, C-3, C-4 , C-16	/	H-13
26	1.16 (s, 3H)	19.3q	C-3, C-4, C-5	H-5	H_3-30, H-16
30	0.97 (s, 3H)	17.5q	C-7, C-11, C-12	/	H_3-26
27	/	80.1s	/	/	/
28	1.13 (s, 3H)	23.0q	C-9, C-27, C-29	/	/
29	1.27 (s, 3H)	26.4q	C-9, C-27, C-28	/	H-9, H_3-26
30	0.97 (s, 3H)	17.5q	C-7, C-11, C-12	/	H-24, H_3-29
31	/	170.7s	/	/	/
32	2.09 (s, 3H)	21.3q	C-31	/	/

Table S3. NMR (600 MHz, $CDCl_3$) data for paxilline B (**3**)

No.	δ_H *(mult., J [Hz], int.)*	δ_C	HMBC	^1H-^1H COSY	NOE
1	7.78 (s)	/	C-2, C-18, C-24	/	/
2	/	151.0s	/	/	/
3	/	50.5s	/	/	/
4	/	43.1s	/	/	/
5	2.80 (td, 13.5,4.9)	28.1t	C-4	H-6	/
	1.49 (m)		C-4,	H-6	/
6	2.31 (m)	28.3t	/	H-5	/
	1.95 (m)		/	H-5	/
7	4.84 (m)	72.9d	C-12	H-7	H_3-25
9	4.85 (m)	80.4d	C-7, C-10, C-11	/	H-7
10	/	195.3s	/	/	/
11	5.85 (d, 2.1)	120.1d	C-10, C-12	/	/
12	/	166.1s	/	/	/
13	/	77.5s	/	/	/
14	2.08 (m)	34.4t	/	H-15	/
	1.66 (m)		/	H-15	/
15	2.07 (m)	20.9t	/	H-16	/
16	2.89 (d, 2.8)	49.6d	/	H-15	H_3-26
17	2.91 (d, 6.2)	29.0t	C-2, C-18	/	/
	β 2.62 (m)		C-2, C-18	/	/
18	/	116.9s	/	/	/
19	/	124.4s	/	/	/
20	/	133.1s	/	/	/
21	6.88 (dd, 7.3, 0.8)	119.0d	C-19, C-23, C-32	H-22	/
22	7.03 (dd, 8.1, 7.3)	121.0d	C-20, C-24	H-23	/
23	7.17 (dd, 8.1, 1.0)	109.3d	C-19, C-21	H-22	/

24	/	139.7s	/	/	/
25	1.34 (s, 3H)	16.2q	C-2, C-3, C-4, C-16	/	H-7
26	1.08 (s, 3H)	19.8q	C-3, C-4, C-13	/	H-16
27	/	81.9s	/	/	/
28	1.46 (s, 3H)	22.8q	C-9, C-27	/	/
29	1.68 (s, 3H)	23.8q	C-9, C-27	/	/
30	/	170.8s	/	/	/
31	2.06 (s, 3H)	22.3q	C-30	/	/
32	3.64 (d, 7.2)	32.0t	/	/	/
33	5.43 (m)	123.7d	/	/	/
34	/	131.8s	/	/	/
35	1.78 (s)	18.0q	C-33, C-34	/	/
36	1.77 (s)	25.8q	C-33, C-34	/	/

Table S4. NMR (600 MHz, $CDCl_3$) data for paxilline C (**4**)

No	δ_H *(mult., J [Hz], int.)*	δ_c	HMBC	^1H-^1H COSY	NOE
1	7.70 (s)	/	C-2, C-18, C-19, C-24	/	/
2	/	151.9s	/	/	/
3	/	50.7s	/	/	/
4	/	43.1s	/	/	/
5	2.78 (td, 13.7, 4.4)	28.0t	/	H-6	/
	1.47 (m)		/	H-6	/
6	2.31 (m)	28.3t	/	H-5, H-7	/
	1.93 (m)		/	H-5, H-7	/
7	4.84 (m)	72.9d	/	H-6	H-25
9	4.85 (brs)	80.4d	/	/	H-25
10	/	195.3s	/	/	/
11	5.84 (d, 2.0)	120.1d	C-7, C-13	/	/
12	/	166.1s	/	/	/
13	/	77.5s	/	/	/
14	2.07 (m)	34.4t	/	H-15	/
	1.67 (m)		/	H-15	/
15	2.05 (m)	20.9t	/	H-14	/
	1.80 (m)		/	/	/
16	2.85 (m)	49.6d	/	H-17	H-26
17	2.74 (dd, 13.0, 6.3)	27.2t	C-2, C-18	H-16	/
	2.45 (dd, 13.2, 10.9)		C-3, C-18	/	/
18	/	117.1s	/	/	/
19	/	125.6s	/	/	/
20	7.25 (brs)	117.6d	C-21, C-32, C-24	/	/
21	/	133.3s	/	/	/
22	6.94 (dd, 8.1, 1.7)	121.4d	C-20 ,C-32	H-23	/

23	7.23 (dd, 8.1, 1.0)	111.3d	C-19, C-21	H-22	/
24	/	138.2s	/	/	/
25	1.33 (s)	16.2q	C-2, C-3, C-4, C-16	/	H-6, H-9
26	1.05 (s)	19.7q	C-3, C-4, C-5, C-13	/	H-16
27	/	81.9s	/	/	/
28	1.46 (s)	22.8q	C-9, C-27	/	/
29	1.68 (s)	23.8q	C-9, C-27, C-30	/	/
30	/	170.8s	/	/	/
31	2.06 (s)	22.3q	C-30	/	/
32	3.43 (d, 7.3)	34.5t	C-20, C-22	H-33	/
33	5.40 (m)	124.6d	/	H-32	/
34	/	131.4s	/	/	/
35	1.77 (s)	17.8q	C-33, C-34	/	/
36	1.76 (s)	25.8q	C-33, C-34	/	/

Table S5. NMR (600 MHz, $CDCl_3$) data for paxilline D (**5**)

No.	δ_H *(mult., J [Hz], int.)*	δ_c	*HMBC*	*^1H-^1H COSY*	*^1H-^1H NOE*
1	7.79 (s)	/	C-20, C-19, C-24, C-2	/	H-23, H-5β
2	/	149.3s	/	/	/
3	/	50.5s	/	/	/
4	/	42.4s	/	/	/
5	2.15 (td, 13.7, 4.0)	31.4t	C-26	H-6, H-17	/
	1.58 (m)		C-7	H_2-15	/
6	2.43 (m)	28.8t	C-31, C-4, C-7	H-5	/
	1.90 (dd, 14.6, 4.3)		/	/	
7	/	96.5s	/	/	/
9	4.06 (s)	76.9d	C-15, C-7, C-10, C-27	/	H_3-31
10	/	198.5s	/	/	/
11	5.81 (d, 2.0)	122.1d	C-7, C-13	H-13	/
12	/	165.1s	/	/	/
13	2.83 (m)	42.0d	C-12, C-4, C-26, C-11, C-14	H-14β	H_3-25
14	1.72 (m)	25.5t	C-16, C-4	H-14β, H-13	/
	1.52 (m)		C-13	H-13	/
15	1.86 (m)	24.1t	C-13, C-3	H-17β, H-13	/
16	2.85 (m)	49.0d	C-17	H-14α	H-6β
17	2.76 (dd, 13.4, 6.4)	27.3t	C-3, C-18, C-2	H-17β	/
	2.44 (m)		/	H-17α	/
18	/	118.4s	/	/	/
19	/	125.0s	/	/	/
20	7.47 (dd, 6.8, 2.2)	118.5d	C-24, C-21	H-21	/
21	7.12 (m)	120.8d	C-19, C-23	H-20	/

22	7.12 (m)	119.8d	C-20, C-24	H-23	
23	7.33 (dd, 6.8, 2.2)	111.4d	C-22, C-19	H-22	
24	/	140.0s	/	/	/
25	1.12 (s)	14.7q	C-2, C-4, C-3	/	H-13, H-6α
26	1.03 (s)	15.9q	C-3, C-5, C-4	/	H-16
27	/	72.4s	/	/	/
28	1.29 (s)	24.0q	C-27, C-29	/	/
29	1.35 (s)	26.7q	C-28, C-27	4.22	/
32	3.40 (s)	49.1q	C-7	/	H-9, H-6α

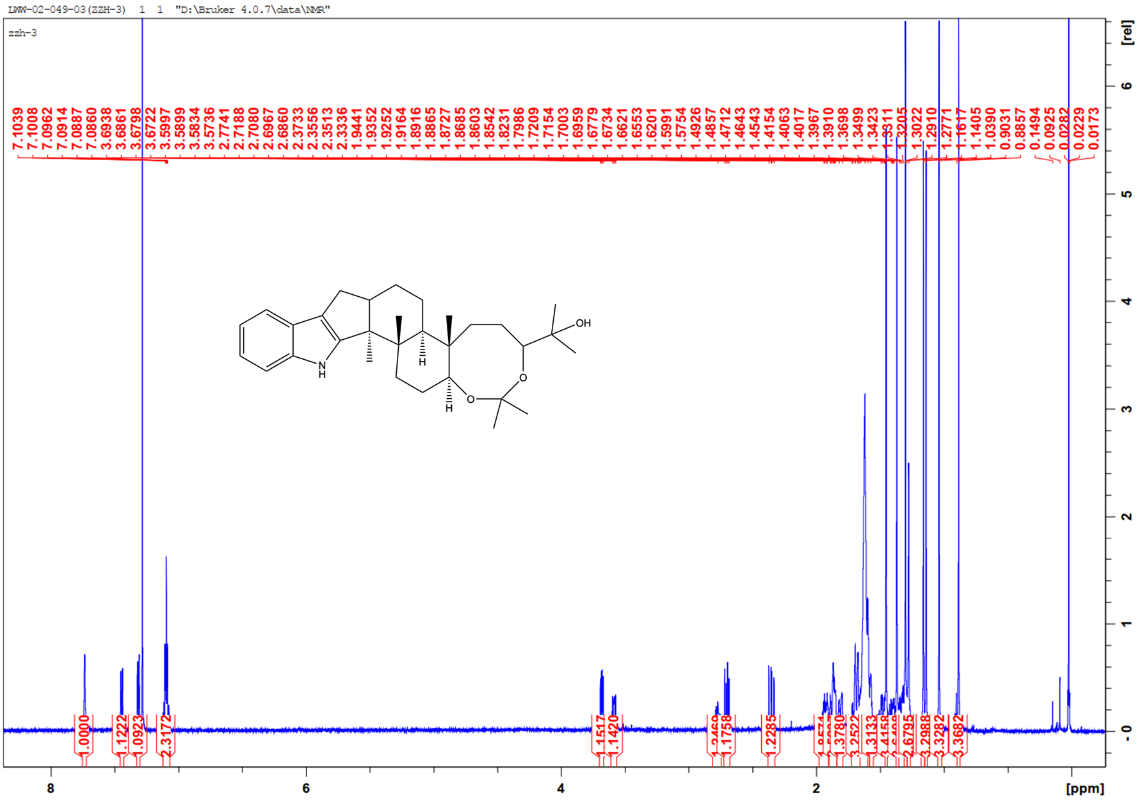

Figure S1. ^{1}H NMR (600 MHz, $CDCl_3$) spectrum of paspaline C (**1**) .

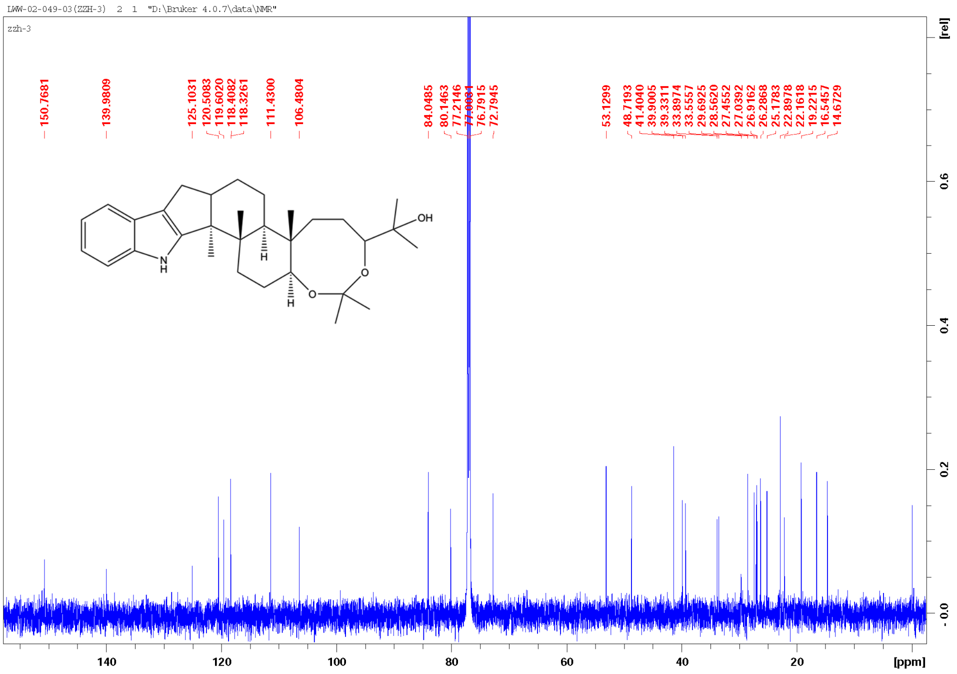

Figure S2. ^{13}C NMR (150 MHz, $CDCl_3$) spectrum of paspaline C (**1**)

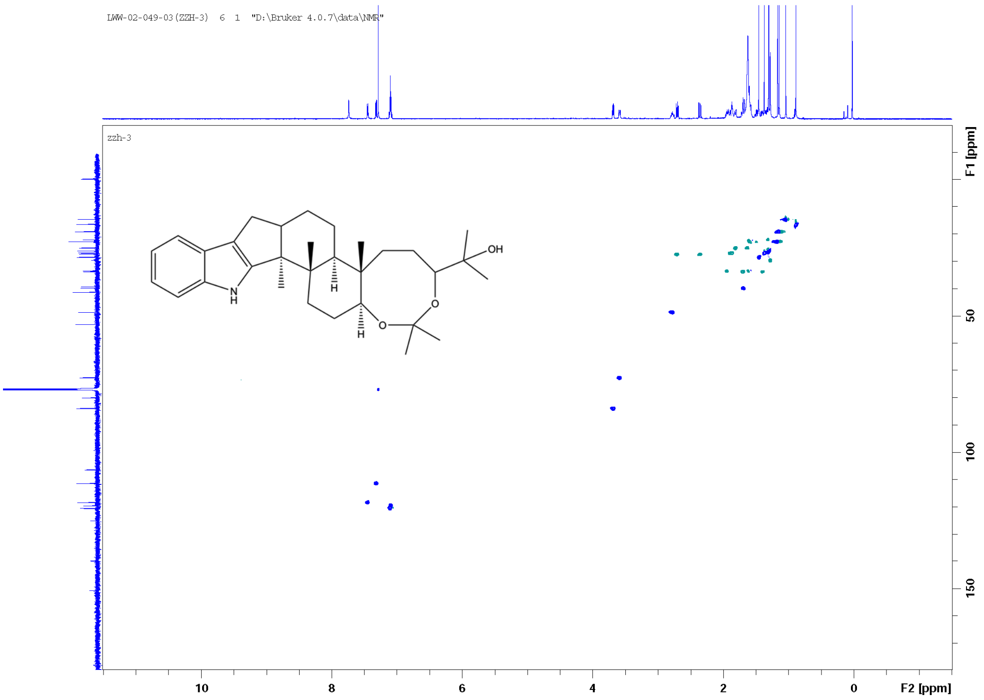

Figure S3. HSQC spectrum of paspaline C (**1**)

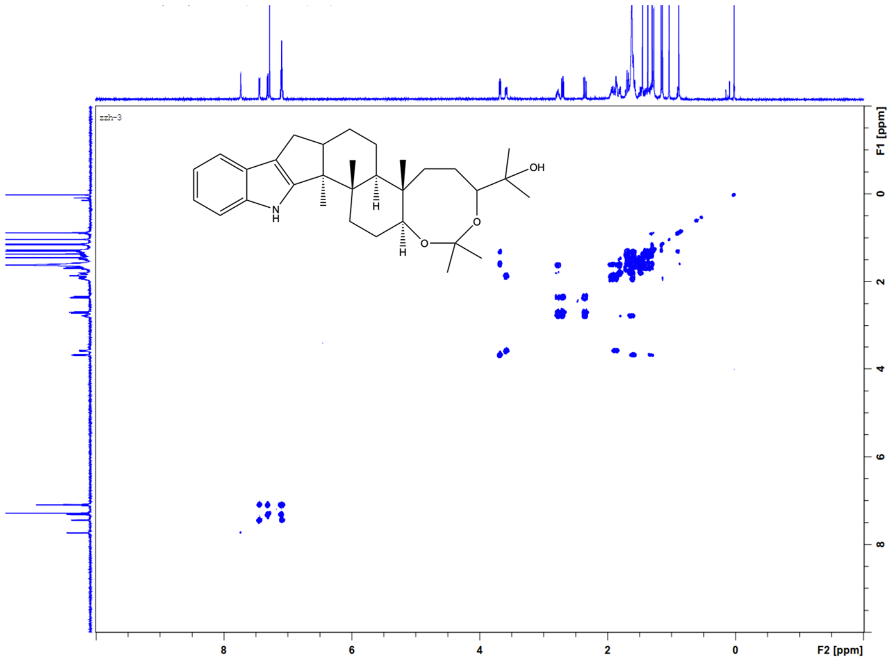

Figure S4. HMBC spectrum of paspaline C (**1**)

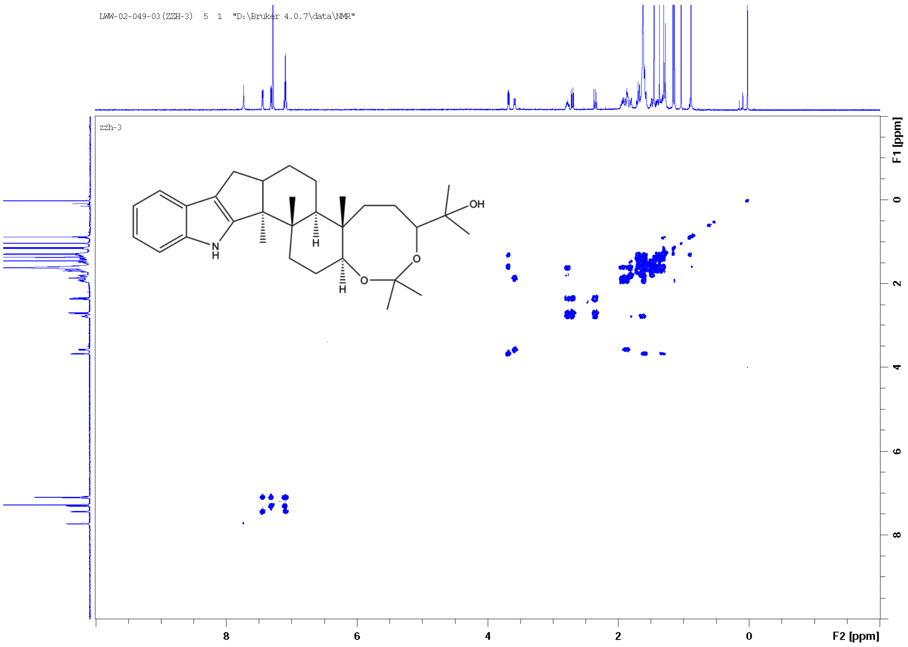

Figure S5. ^{1}H -^{1}H COSY spectrum of paspaline C (**1**)

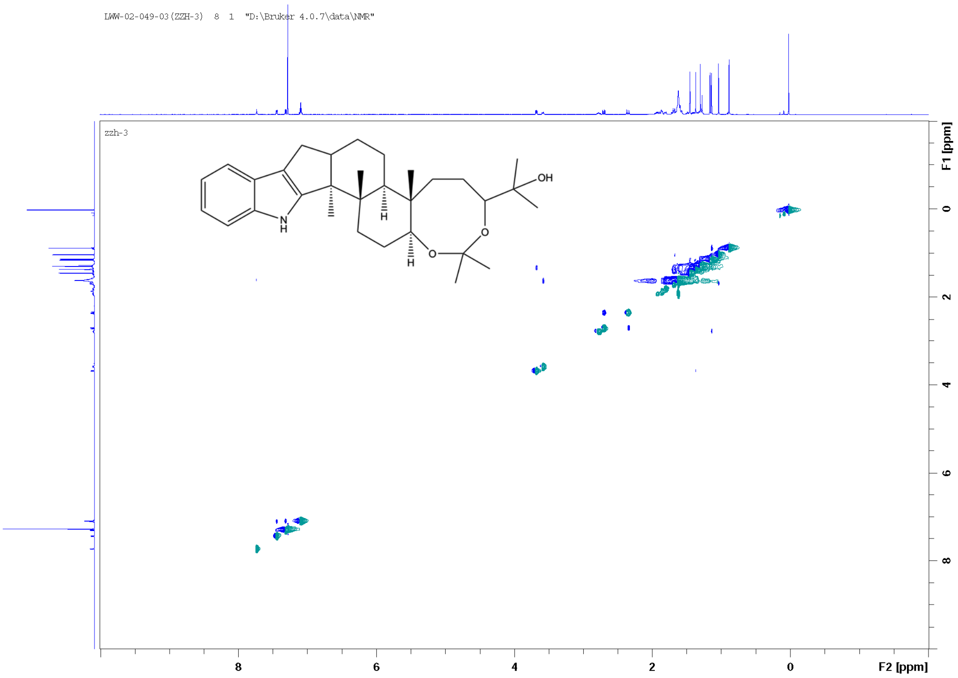

Figure S6. NOE spectrum of paspaline C (**1**)

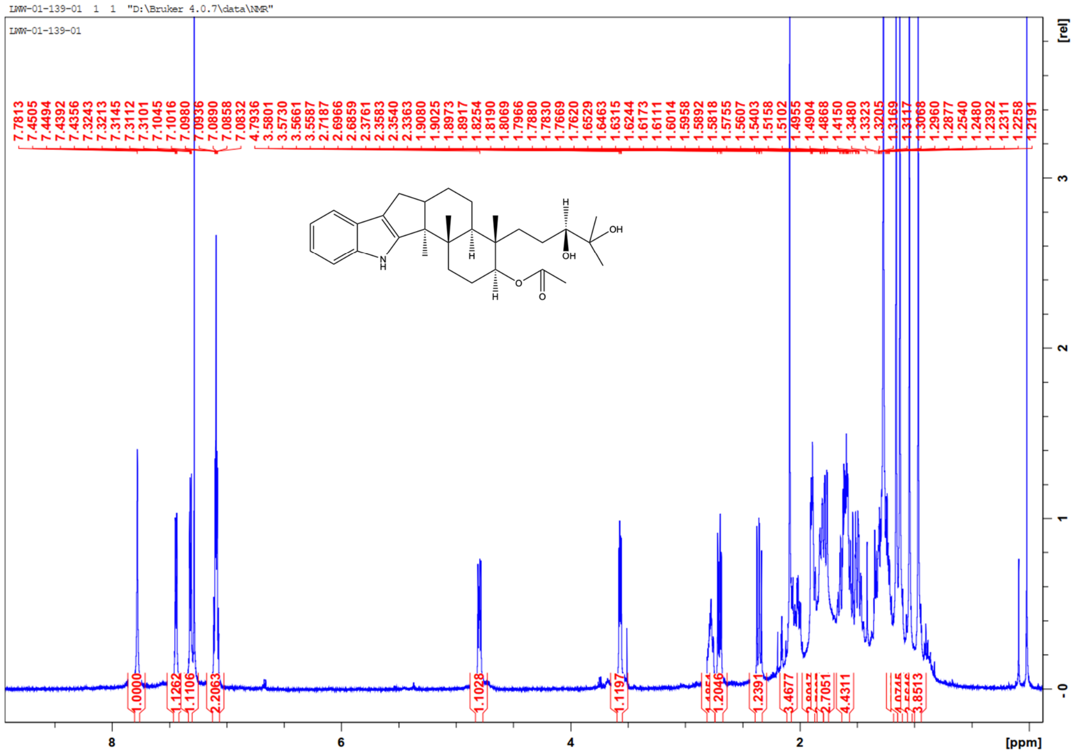

Figure S7. ¹H NMR (600 MHz, $CDCl_3$) spectrum of paspaline D (**2**)

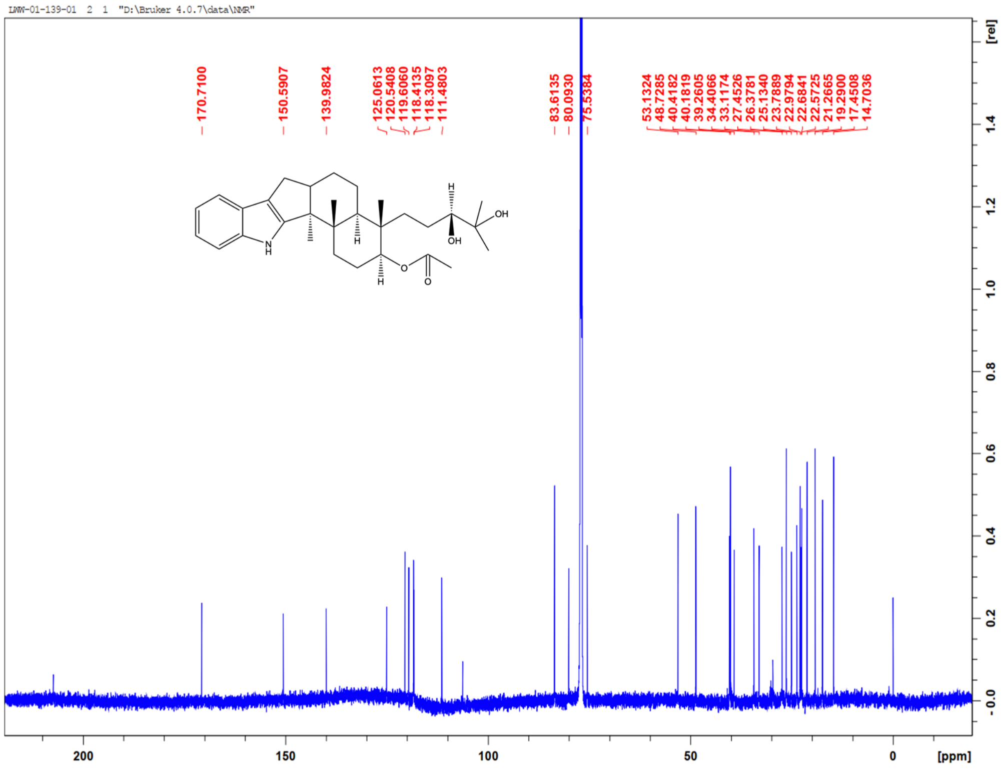

Figure S8. ¹³C NMR (150 MHz, $CDCl_3$) spectrum of paspaline D (**2**)

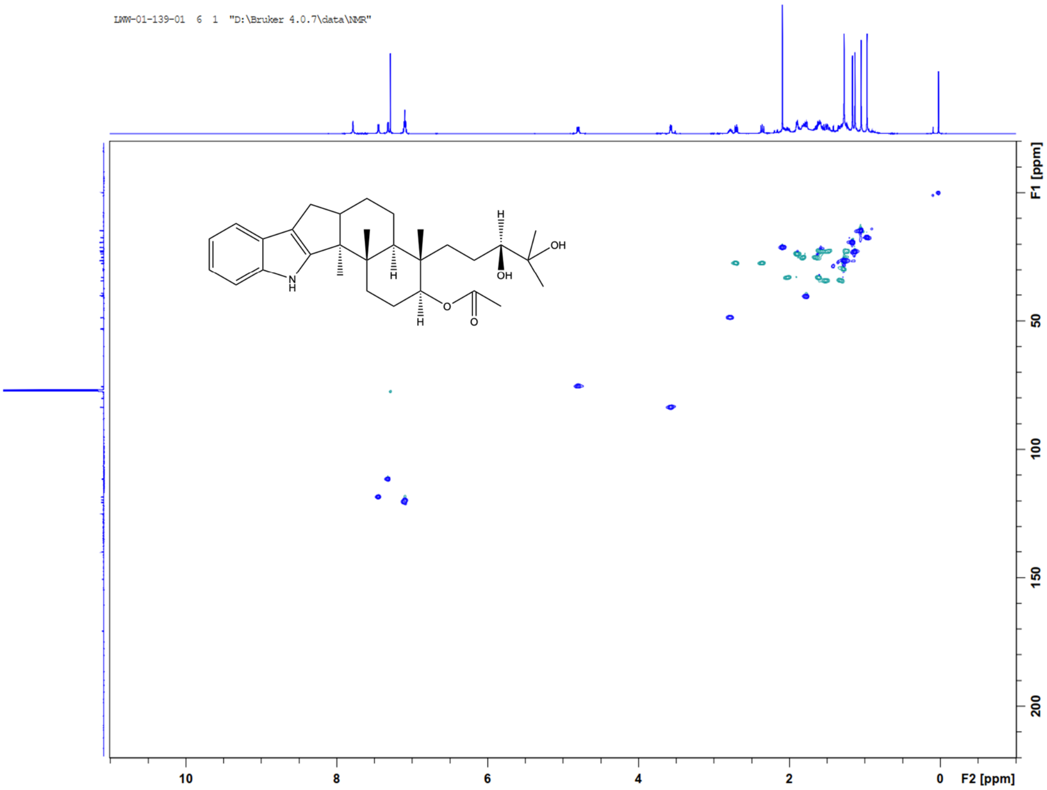

Figure S9. HSQC spectrum of paspaline D (**2**)

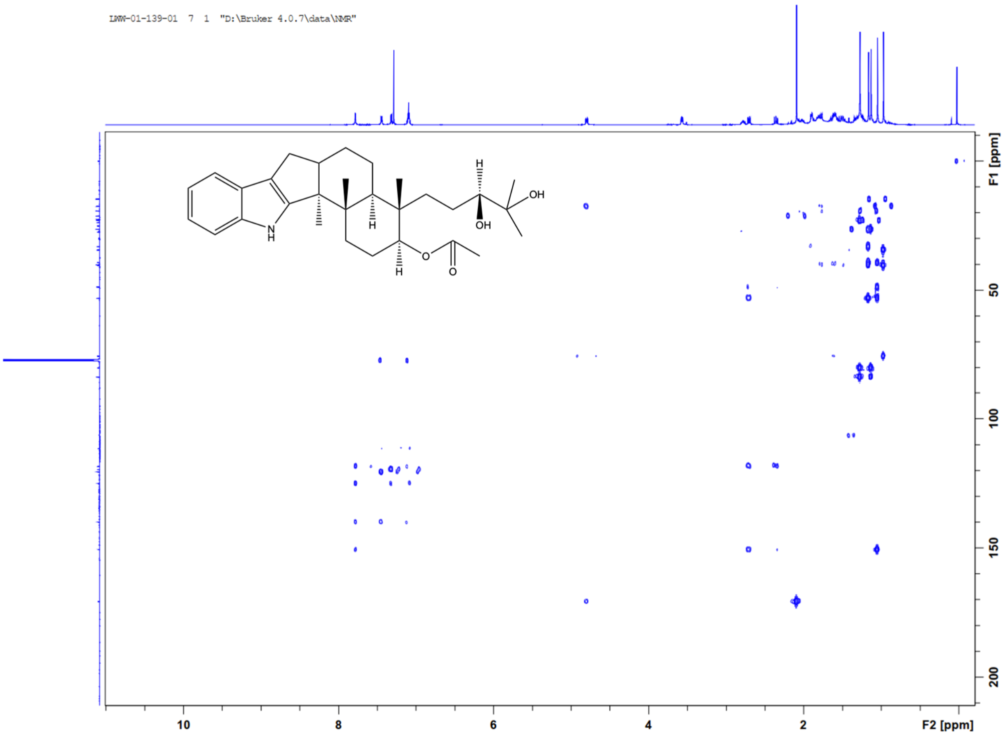

Figure S10. HMBC spectrum of paspaline D (**2**)

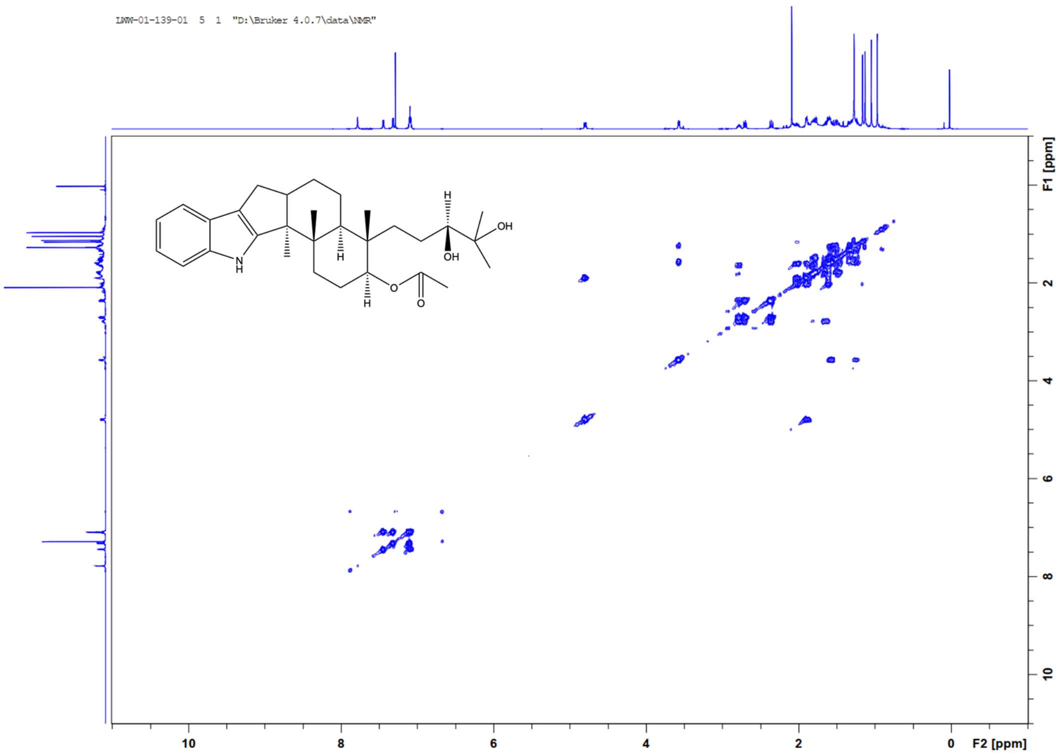

Figure S11. ^{1}H -^{1}H COSY spectrum of paspaline D (**2**)

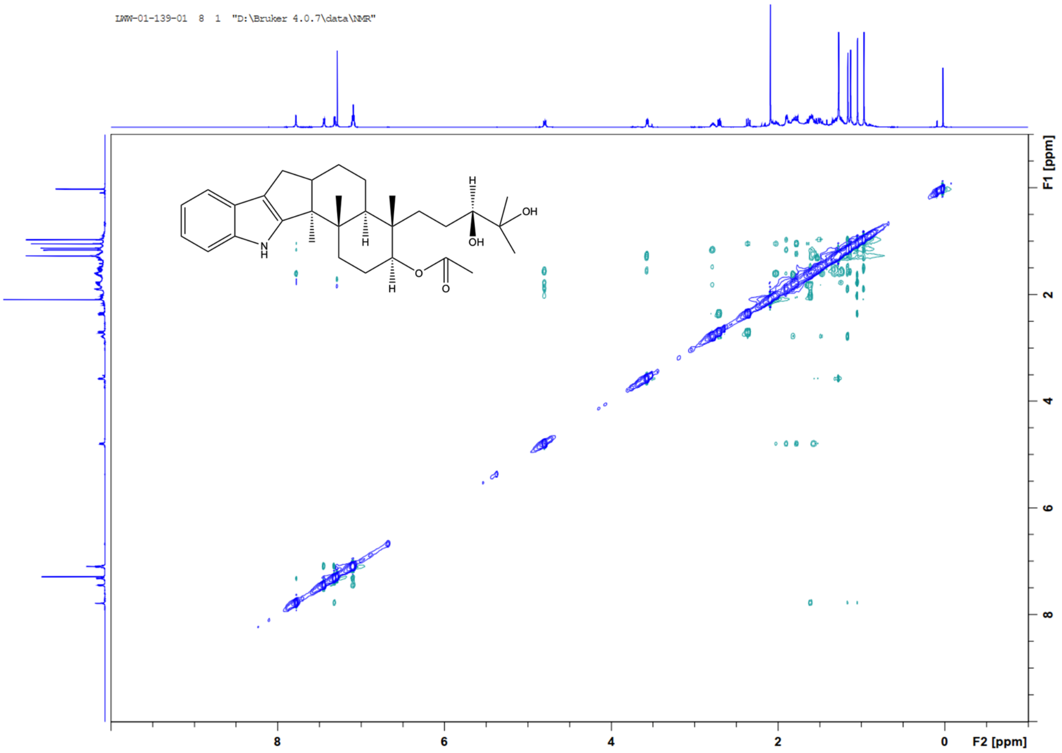

Figure S12. NOE spectrum of paspaline D (**2**)

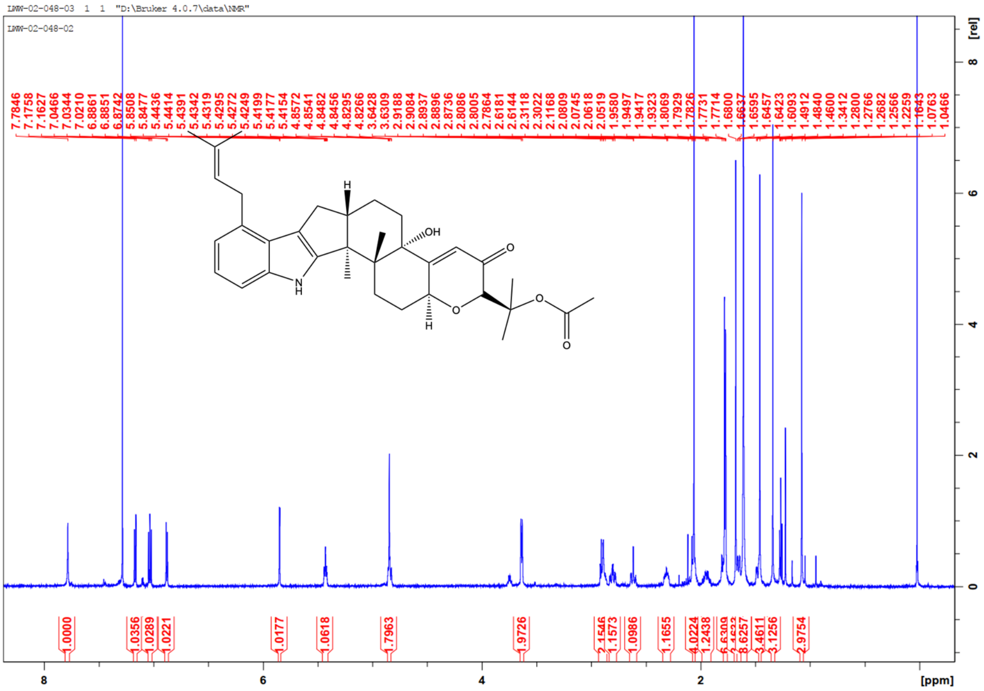

Figure S13. ^{1}H NMR (600 MHz, $CDCl_3$) spectrum of paxilline B (**3**)

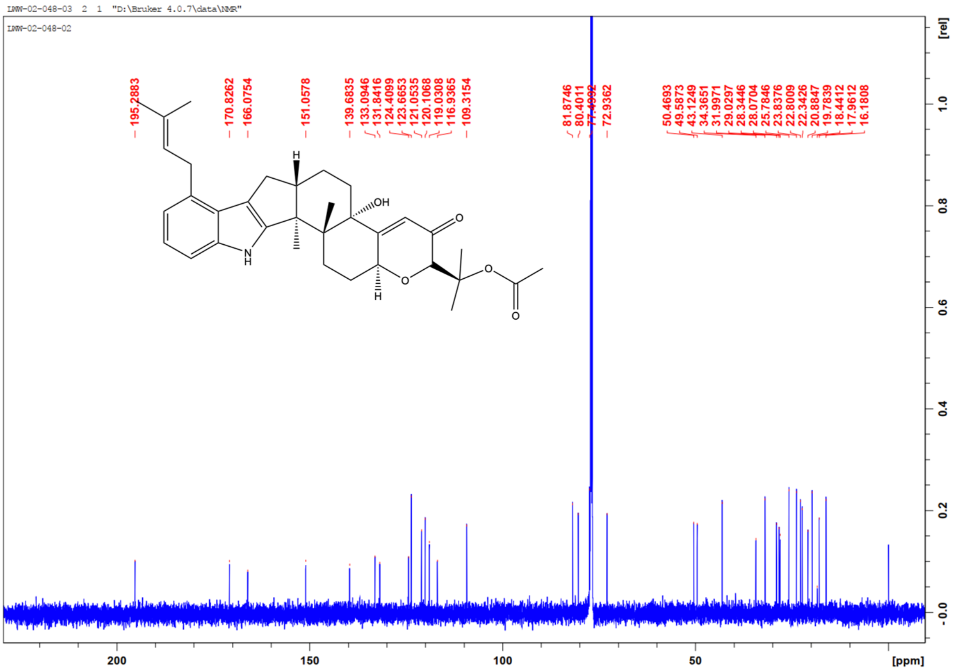

Figure S14. ^{13}C NMR (150 MHz, $CDCl_3$) spectrum of paxilline B (**3**)

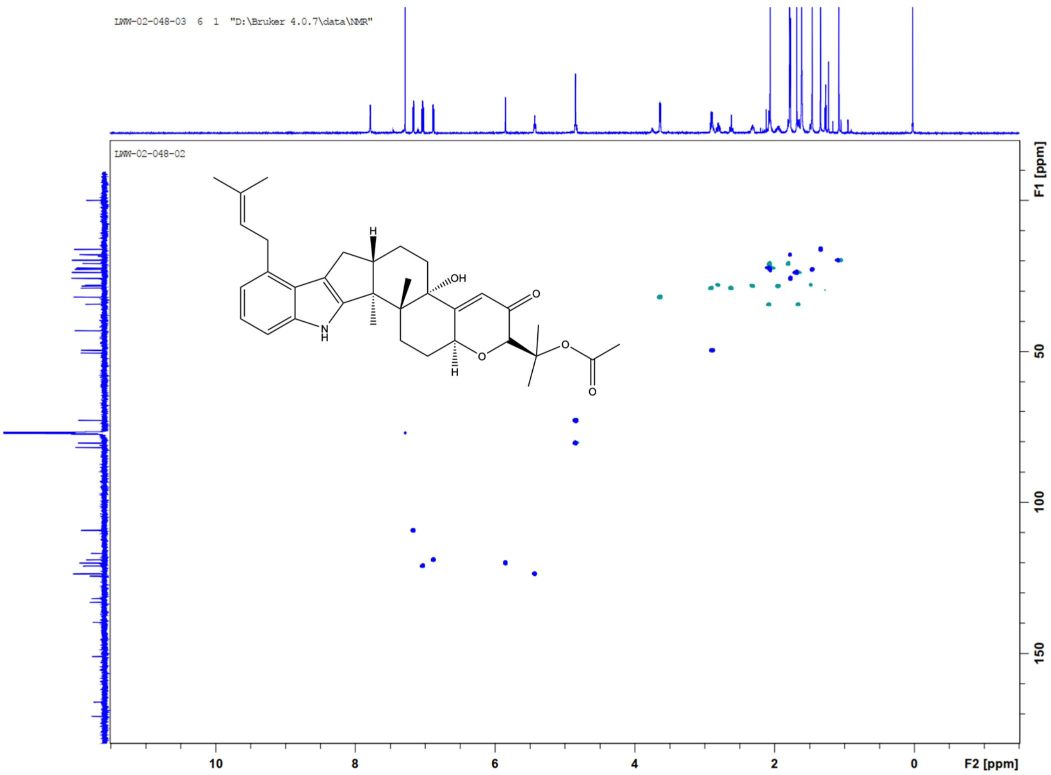

Figure S15. HSQC spectrum of paxilline B (**3**)

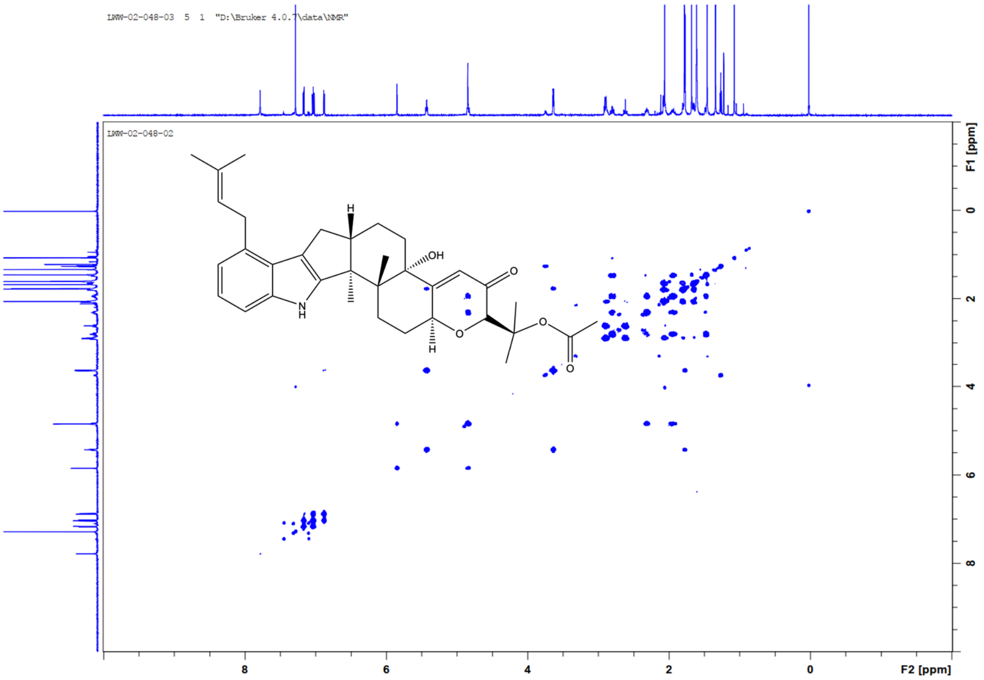

Figure S16. HMBC spectrum of paxilline B (**3**)

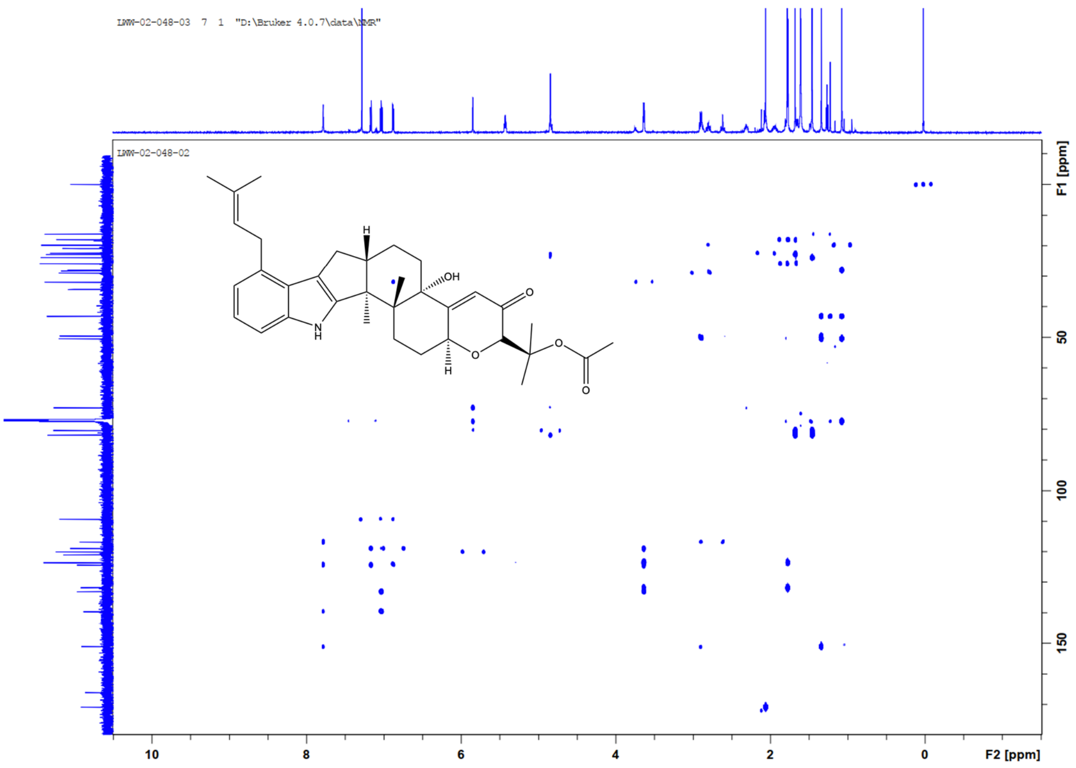

Figure S17. ^{1}H -^{1}H COSY spectrum of paxilline B (**3**)

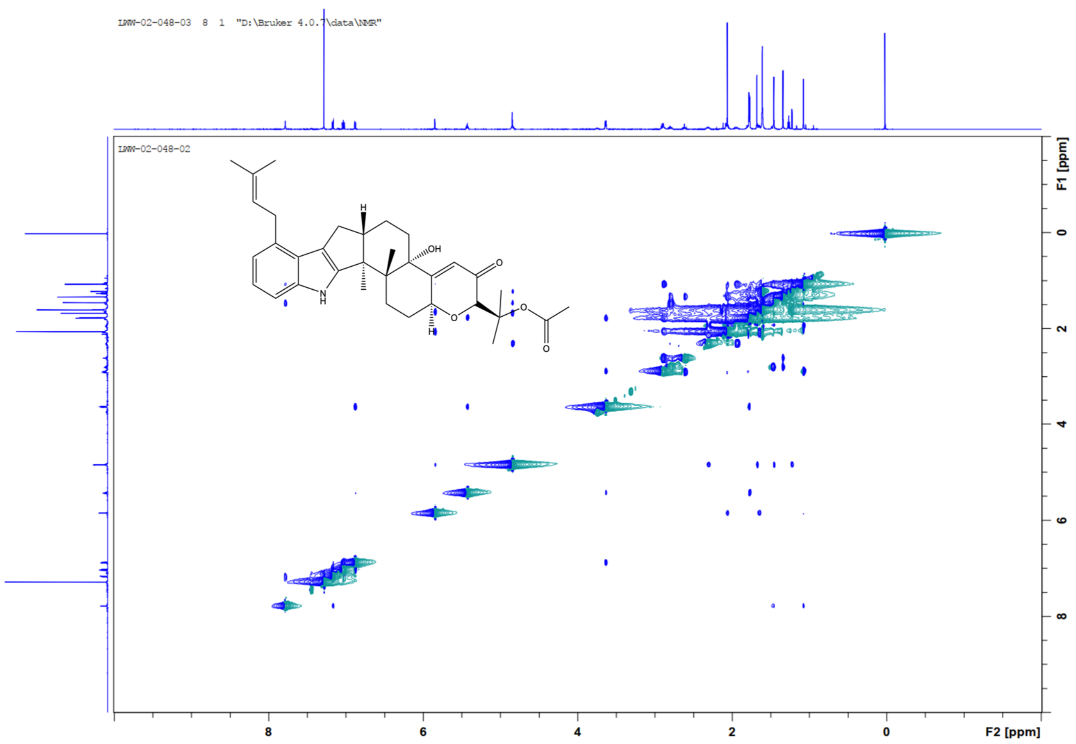

Figure S18. NOE spectrum of paxilline B (**3**)

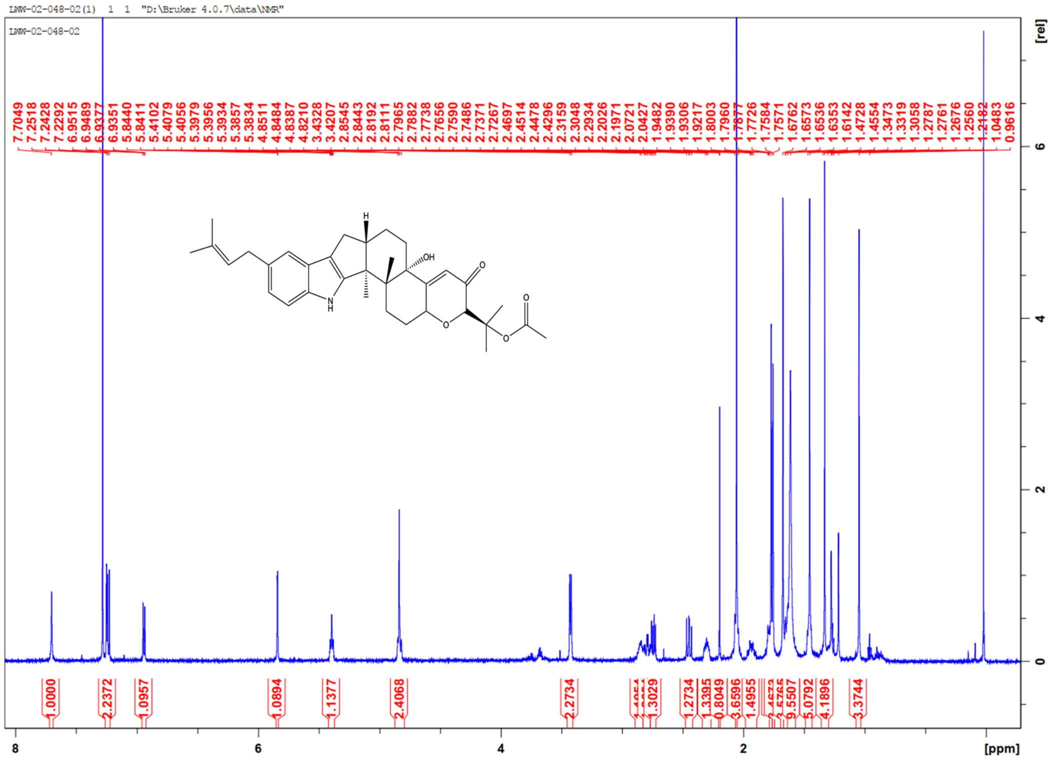

Figure S19. ^{1}H NMR (600 MHz, $CDCl_3$) spectrum of paxilline C (**4**)

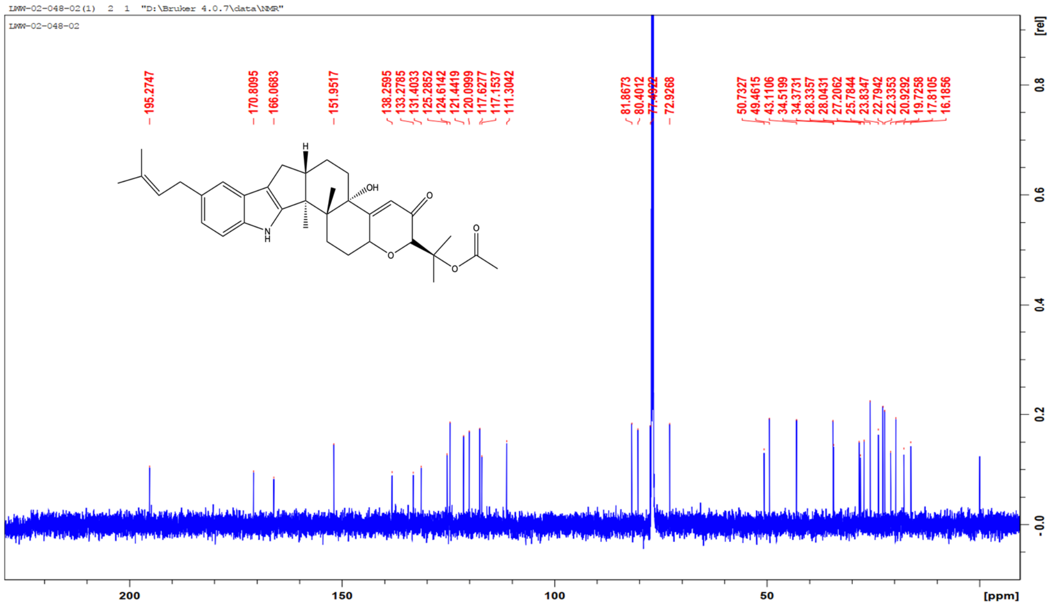

Figure S20. ^{13}C NMR (150 MHz, $CDCl_3$) spectrum of paxilline C (**4**)

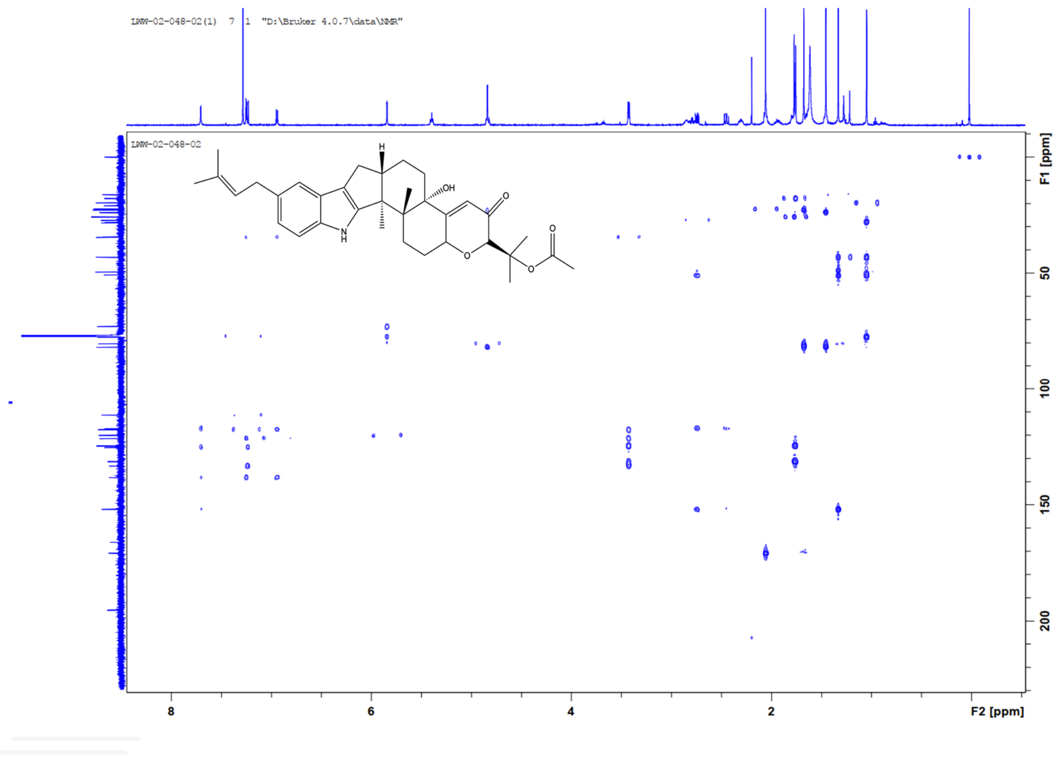

Figure S21. HSQC spectrum of paxilline C (**4**)

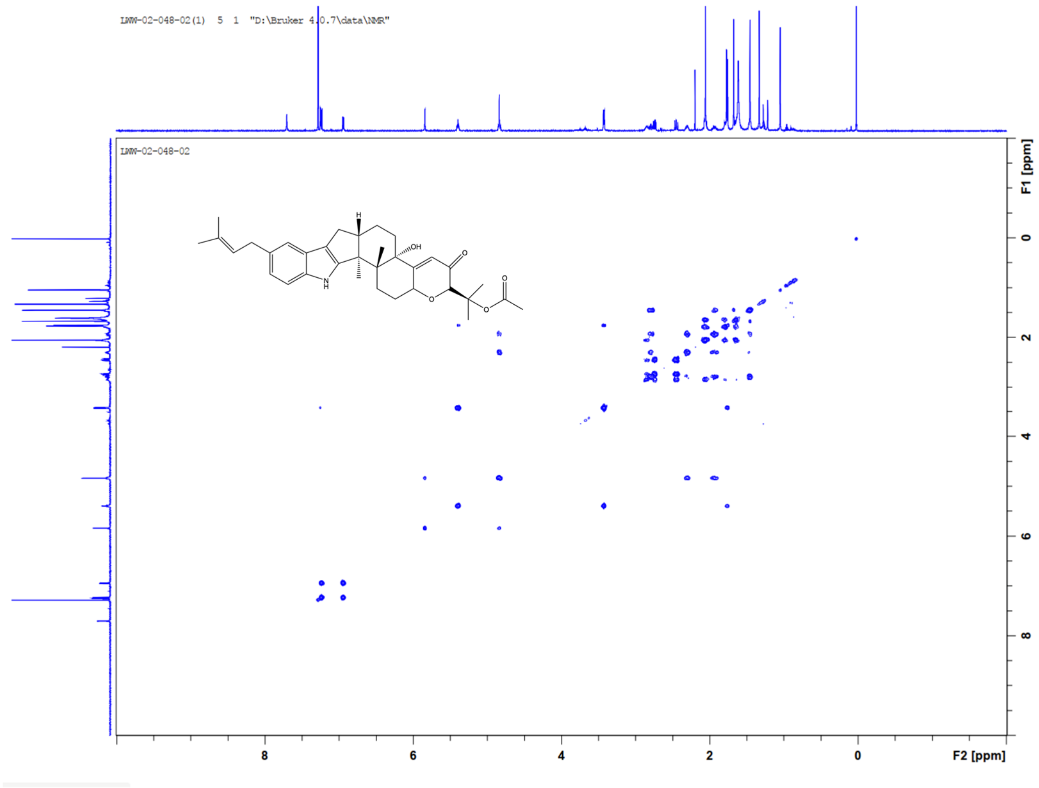

Figure S22. HMBC spectrum of paxilline C (**4**)

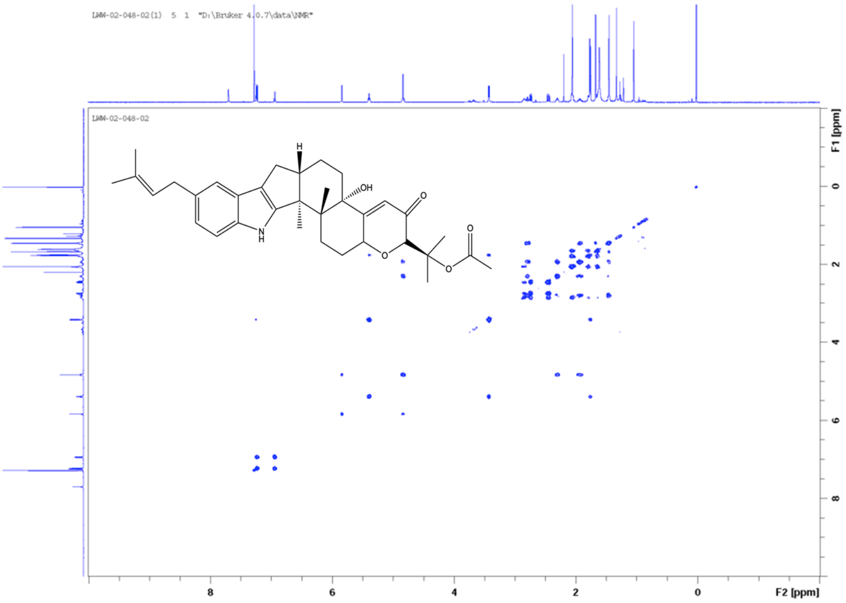

Figure S23. ^{1}H -^{1}H COSY spectrum of paxilline C (**4**)

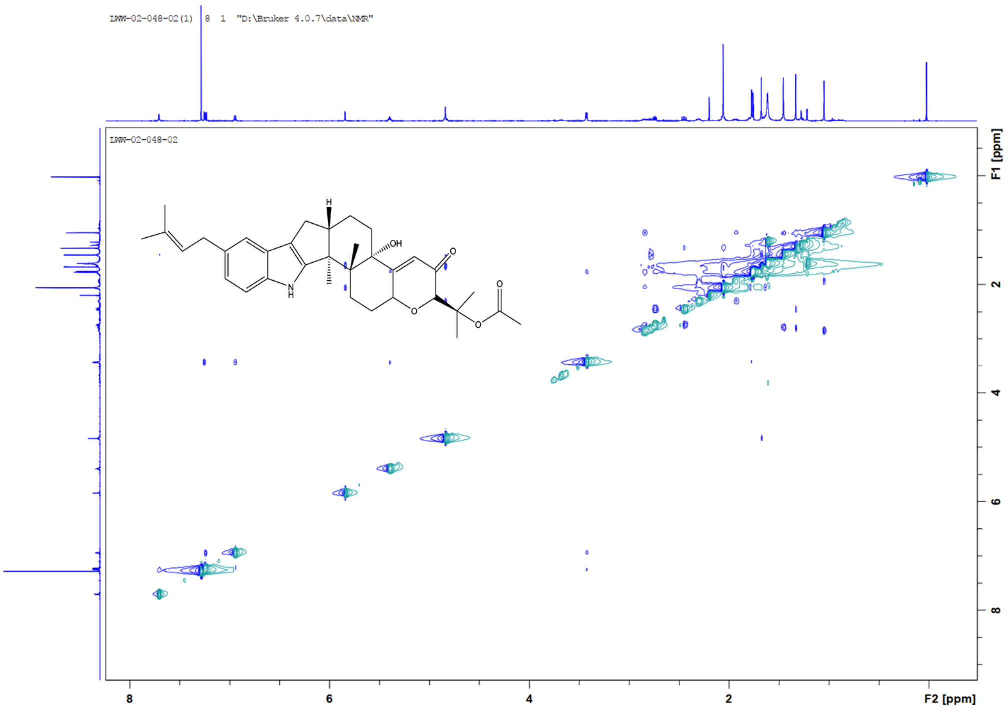

Figure S24. NOE spectrum of paxilline C (**4**)

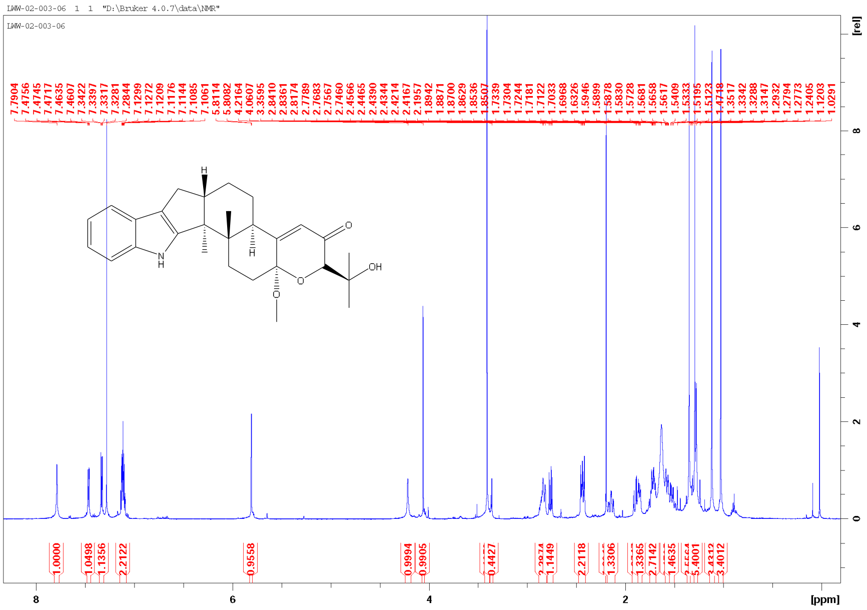

Figure S25. ^{1}H NMR (600 MHz, $CDCl_3$) spectrum of paxilline D (**5**)

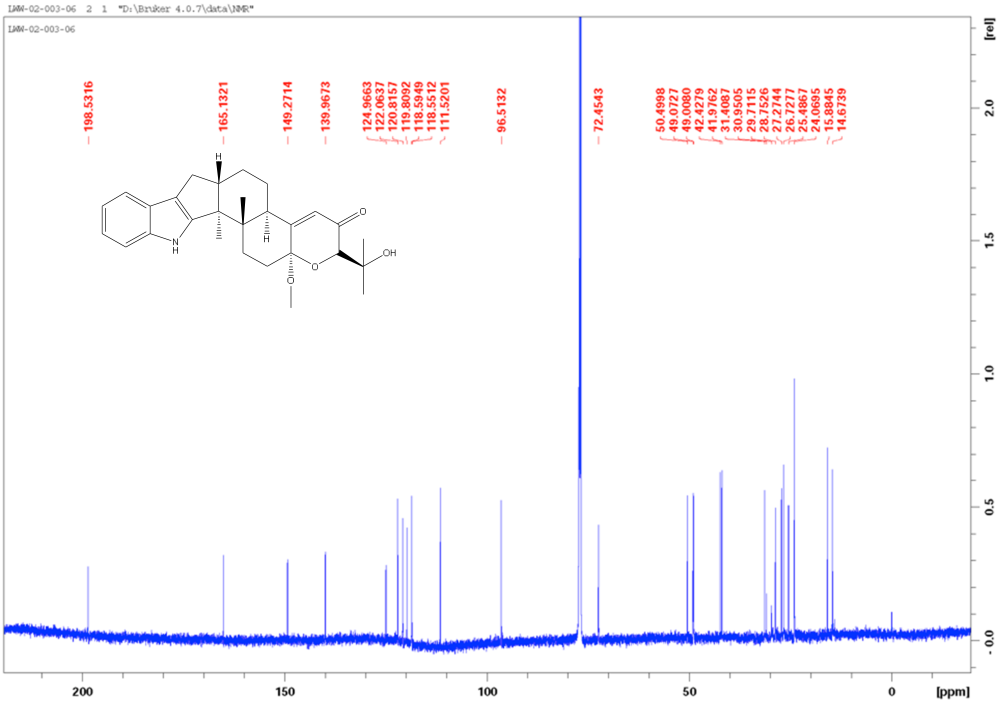

Figure S26. ^{13}C NMR (150 MHz, $CDCl_3$) spectrum of paxilline D (**5**)

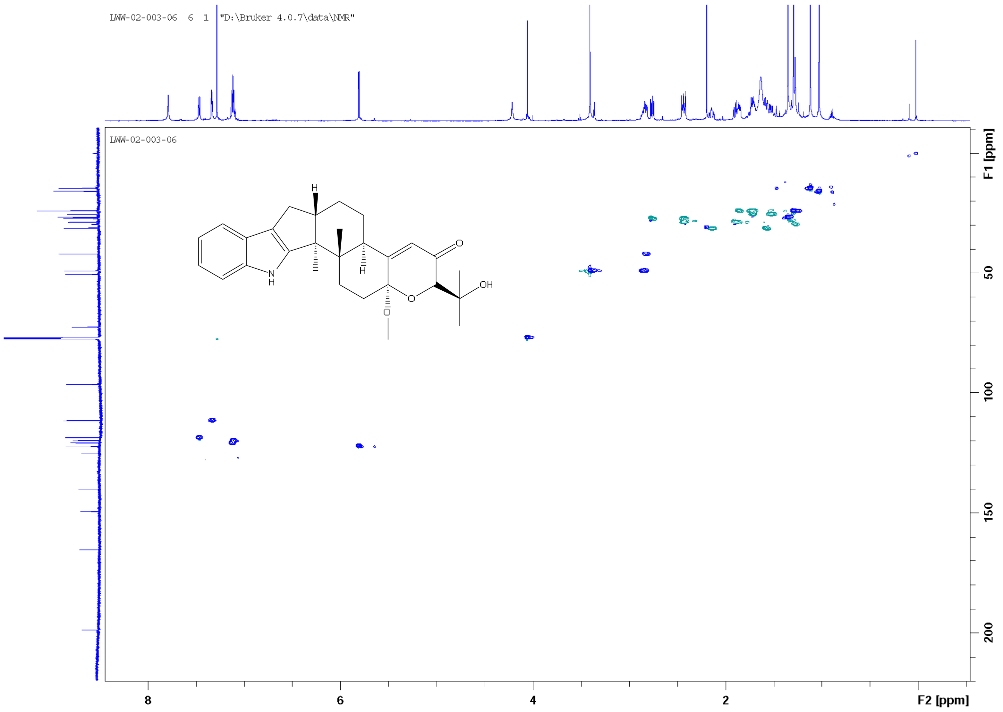

Figure S27. HSQC spectrum of paxilline D (**5**)

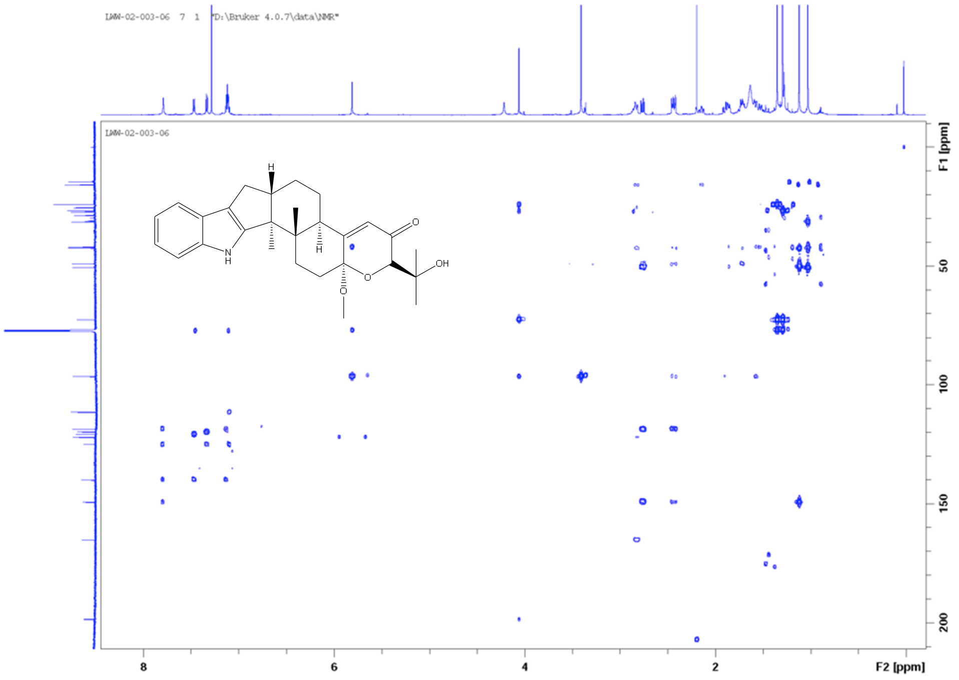

Figure S28. HMBC spectrum of paxilline D (**5**)

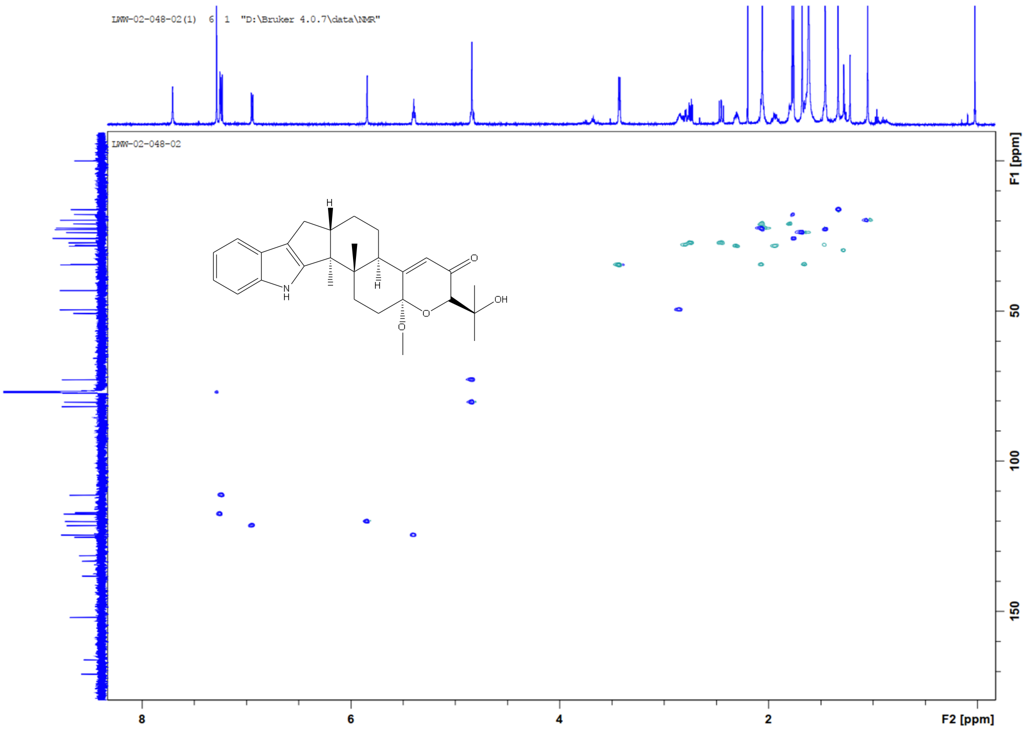

Figure S29. ^{1}H -^{1}H COSY spectrum of paxilline D (**5**)

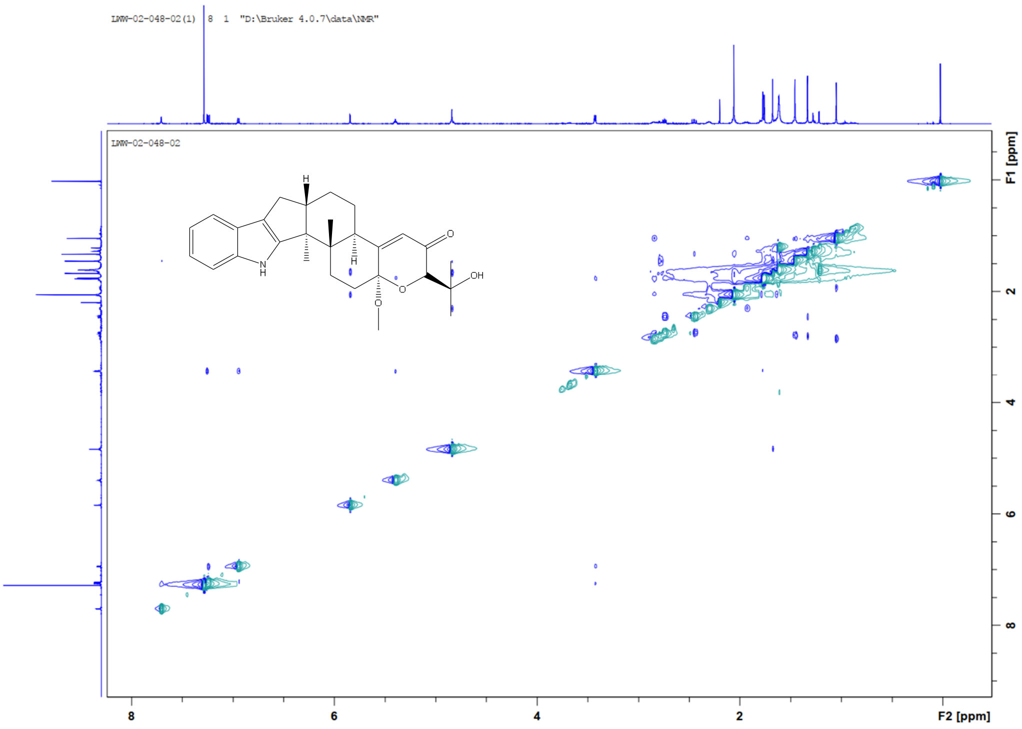

Figure S30. NOE spectrum of paxilline D (**5**)

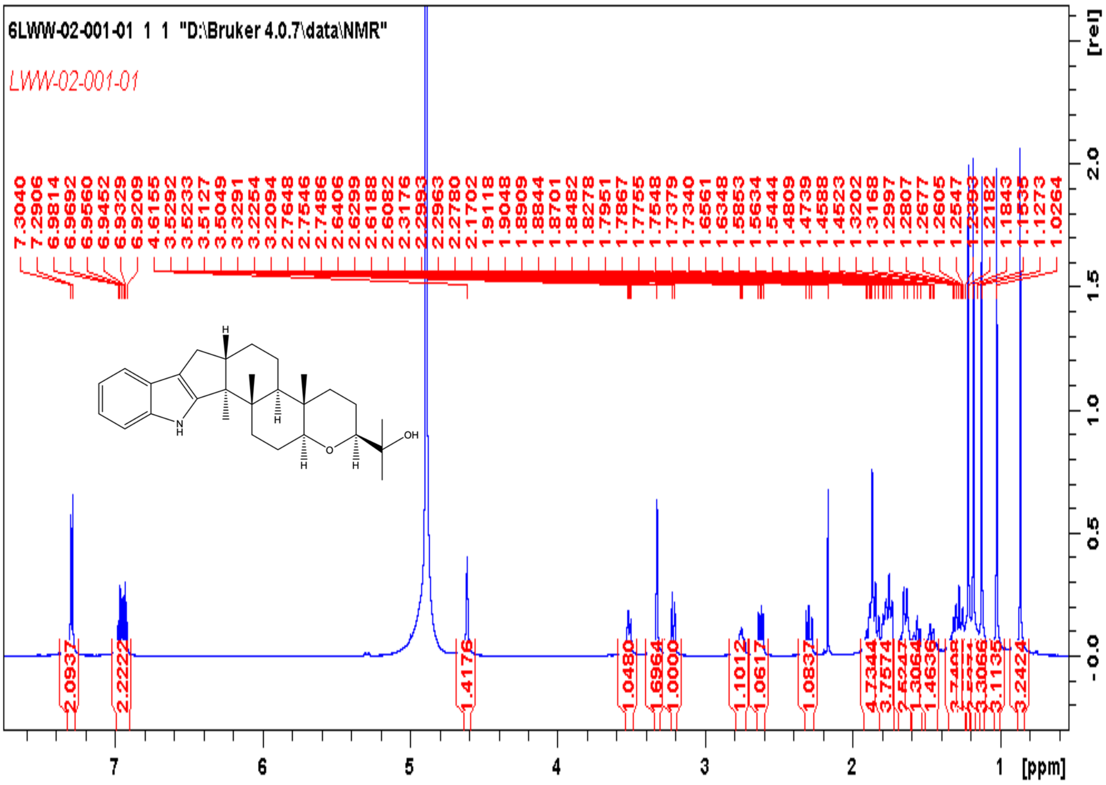

Figure S31. ^{1}H NMR (600 MHz, $CDCl_3$) spectrum of paspaline (**6**)

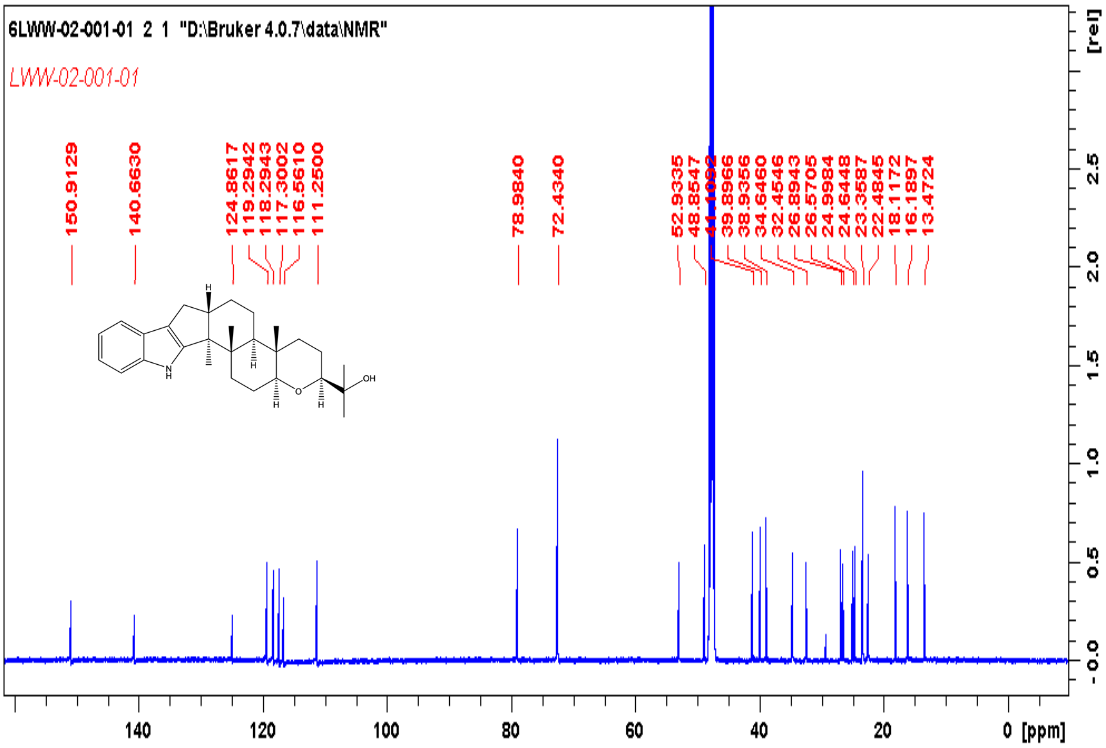

Figure S32. ^{13}C NMR (150 MHz, $CDCl_3$) spectrum of paspaline (**6**)

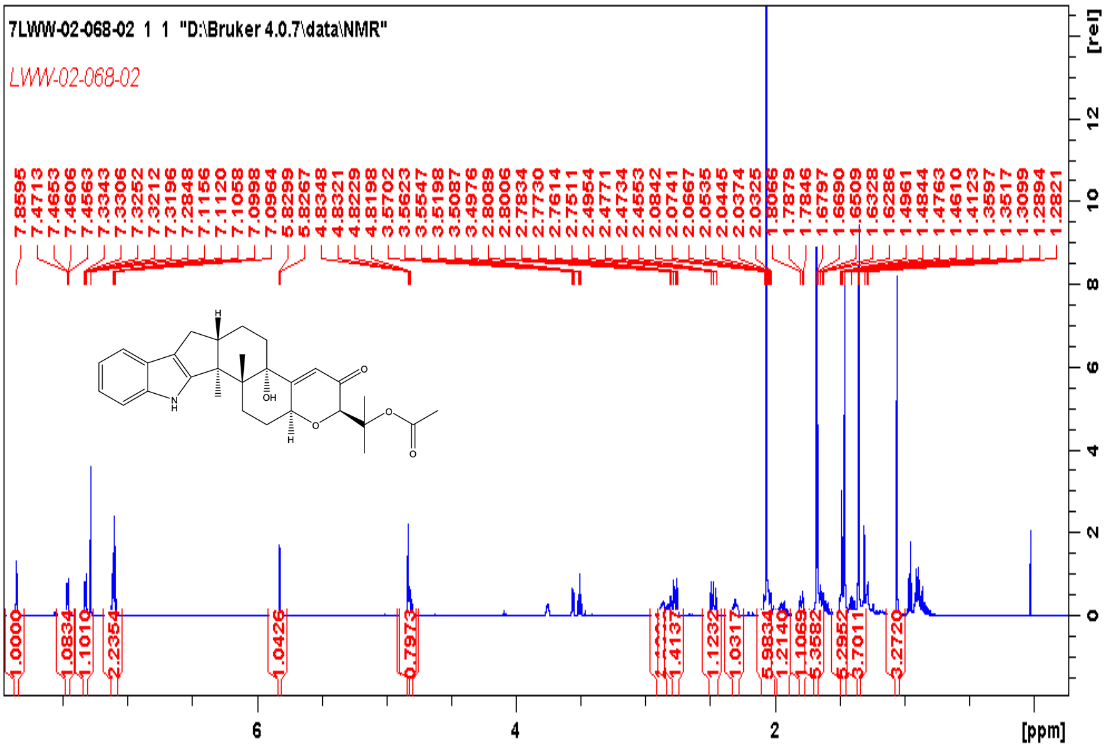

Figure S33. ^{1}H NMR (600 MHz, $CDCl_3$) spectrum of 1′-O-acetyl paxilline (**7**)

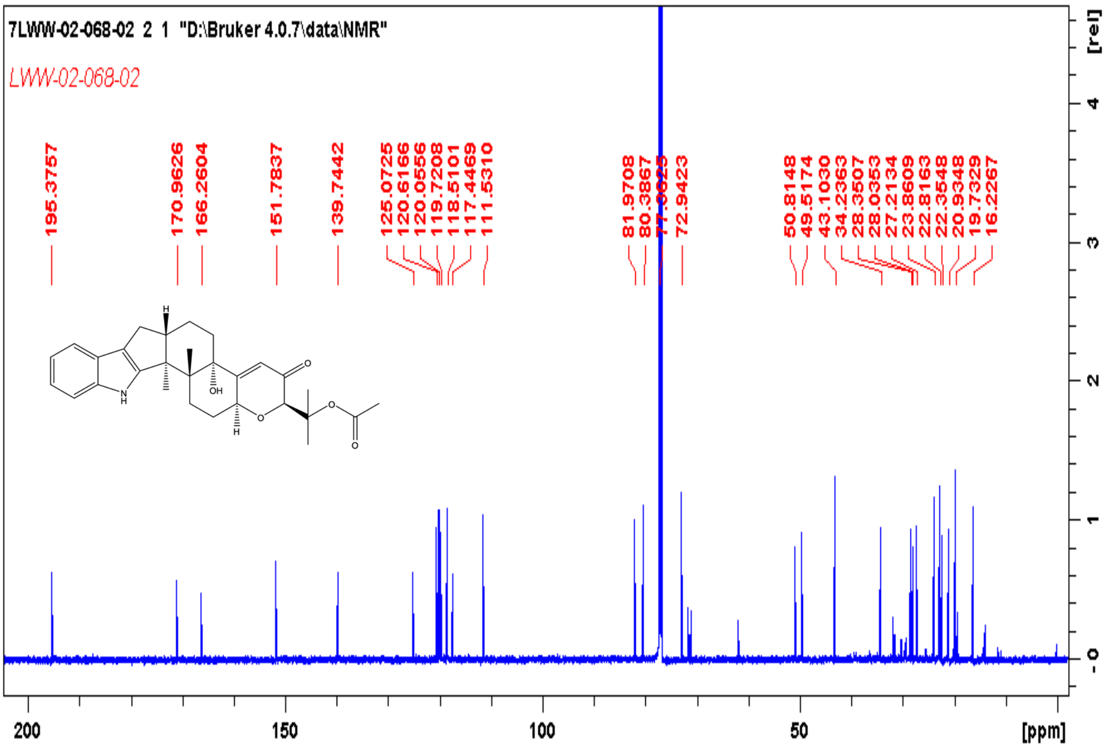

Figure S34. ^{13}C NMR (150 MHz, $CDCl_3$) spectrum of 1′-O-acetyl paxilline (**7**)

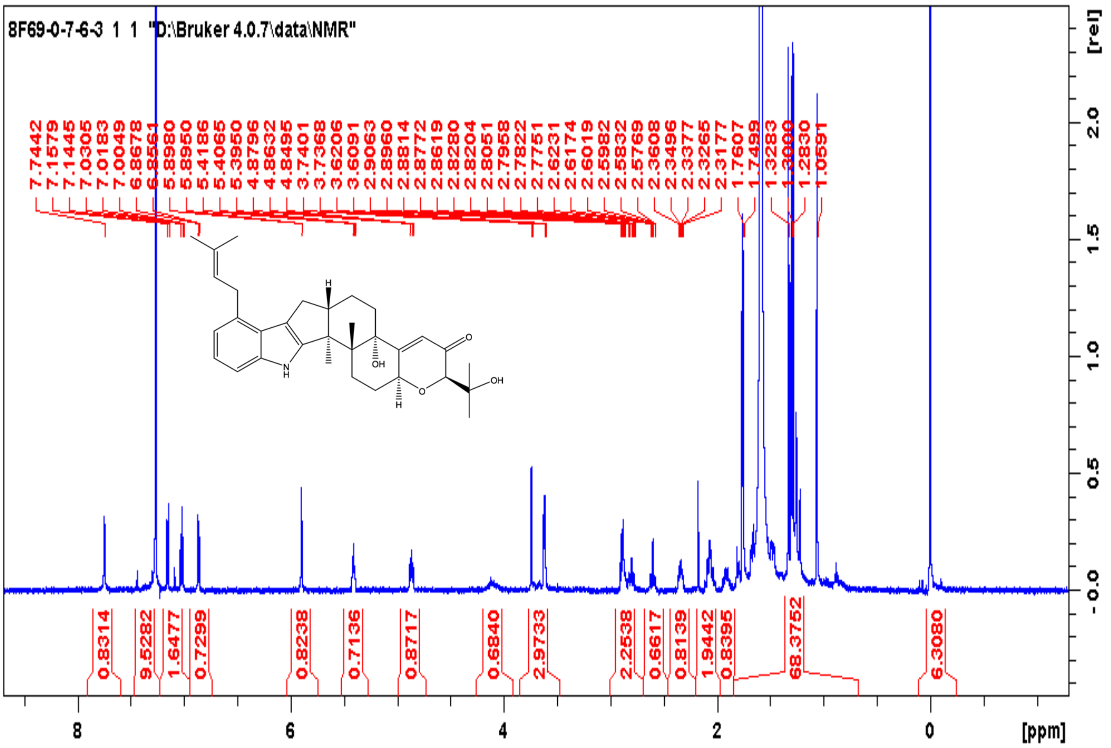

Figure S35. ^{1}H NMR (600 MHz, $CDCl_3$) spectrum of 20-prenylated paxilline (**8**)

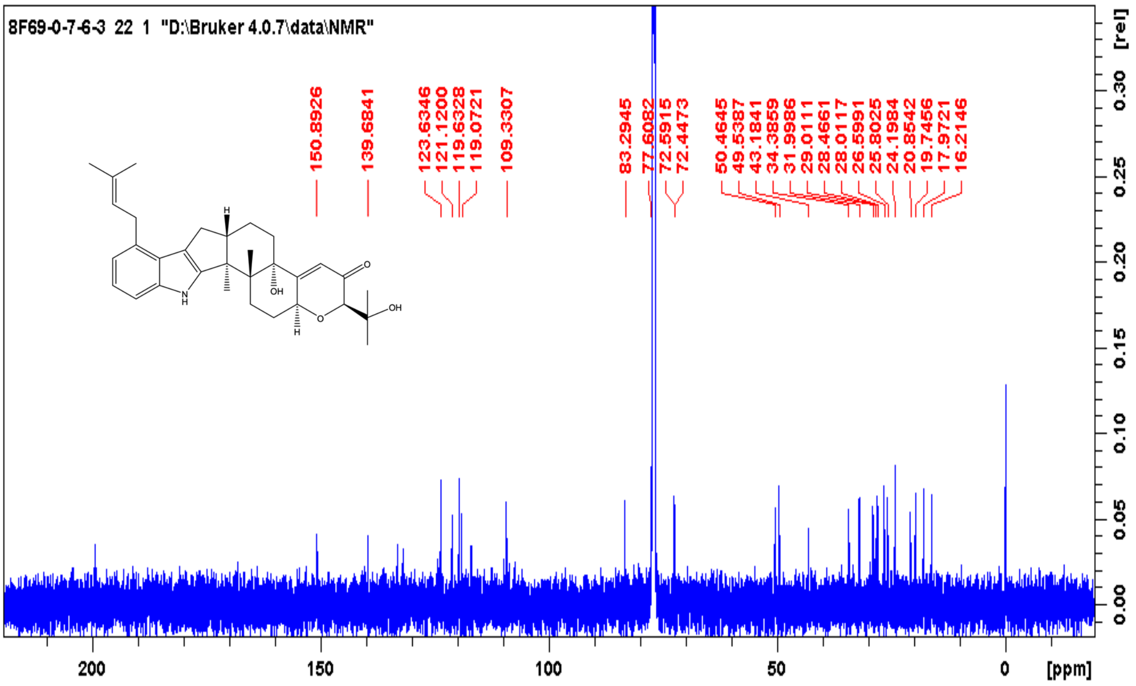

Figure S36. ^{13}C NMR (150 MHz, $CDCl_3$) spectrum of 20-prenylated paxilline (**8**)

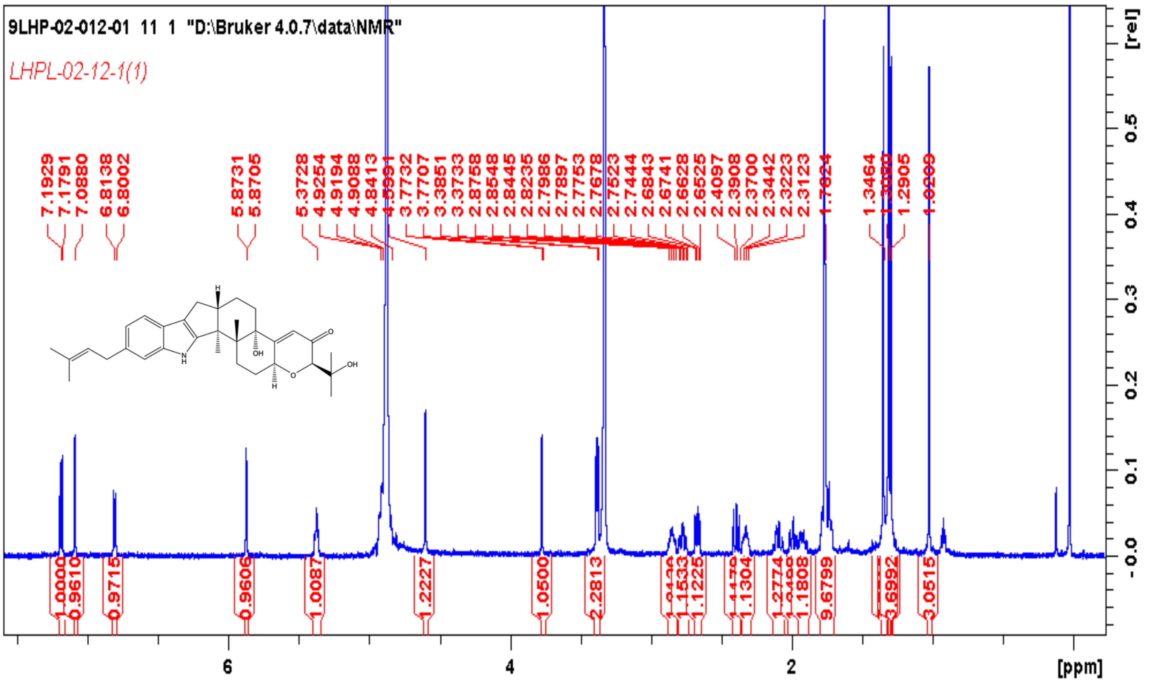

Figure S37. ¹H NMR (600 MHz, $CDCl_3$) spectrum of 22-prenylated paxilline (**9**)

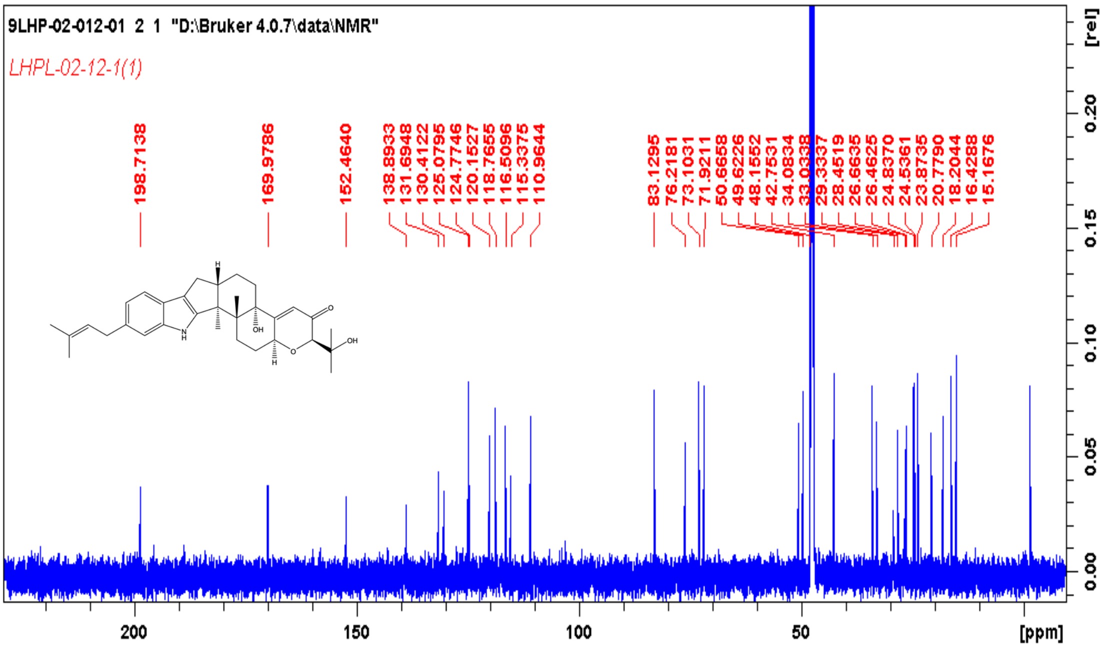

Figure S38. ¹³C NMR (150 MHz, $CDCl_3$) spectrum of 22-prenylated paxilline (**9**)

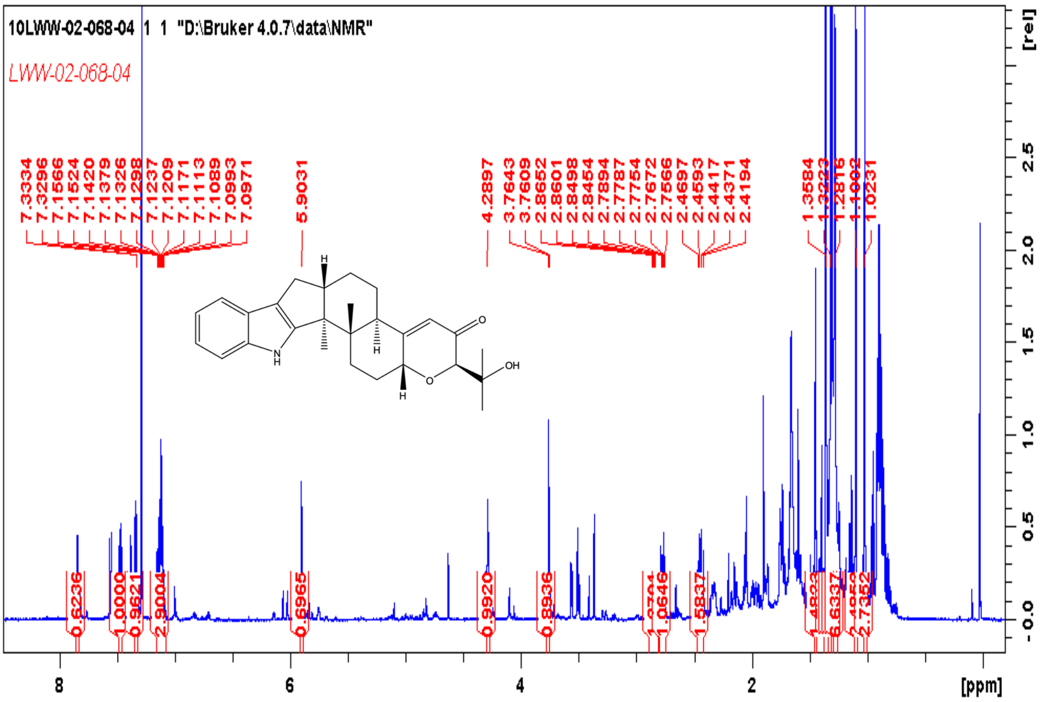

Figure S39. ¹H NMR (600 MHz, $CDCl_3$) spectrum of 13-deoxy-paxilline (**10**)

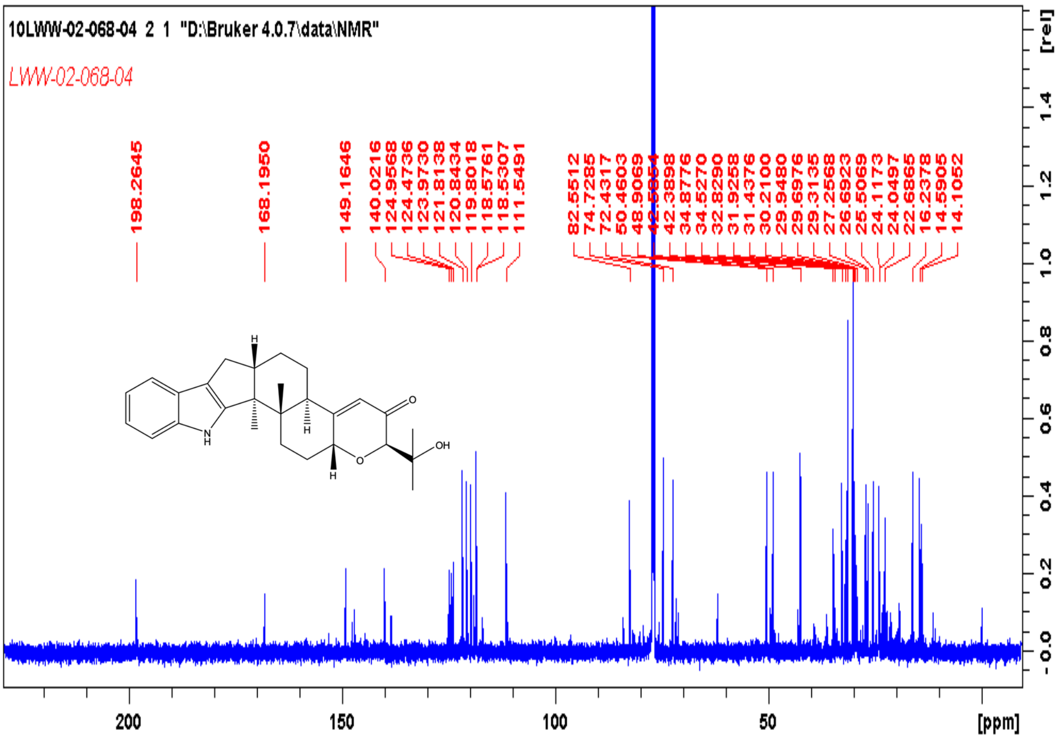

Figure S40. ¹³C NMR (150 MHz, $CDCl_3$) spectrum of 13-deoxy-paxilline (**10**)

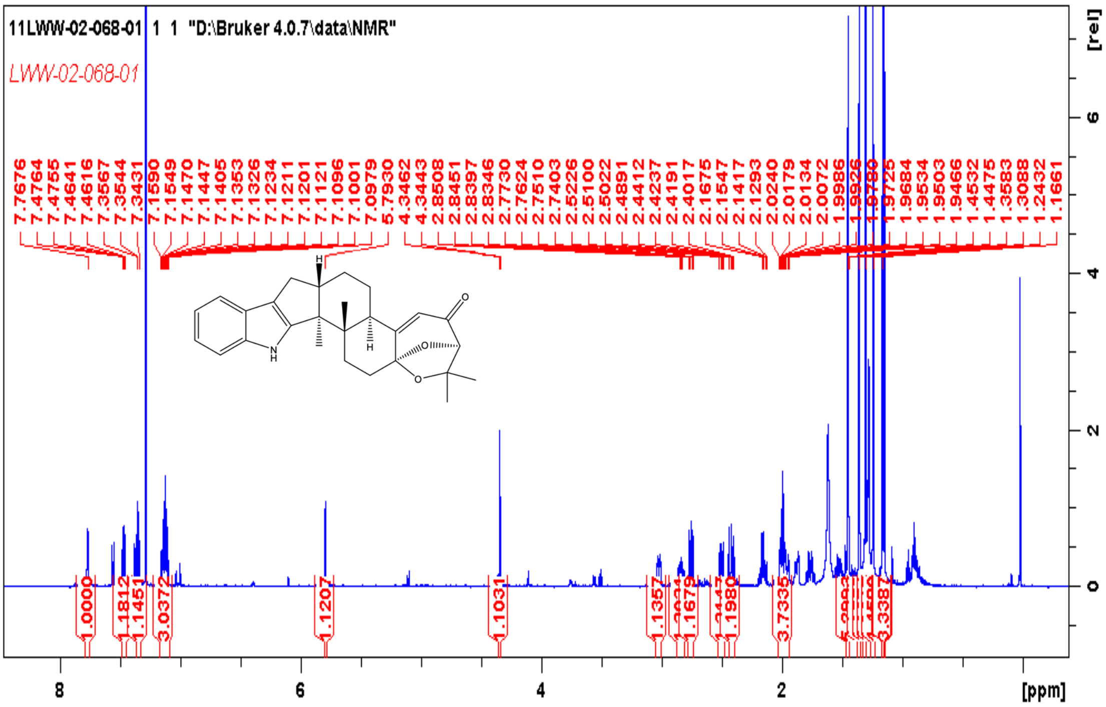

Figure S41. ^{1}H NMR (600 MHz, $CDCl_3$) spectrum of paspalicine (**11**)

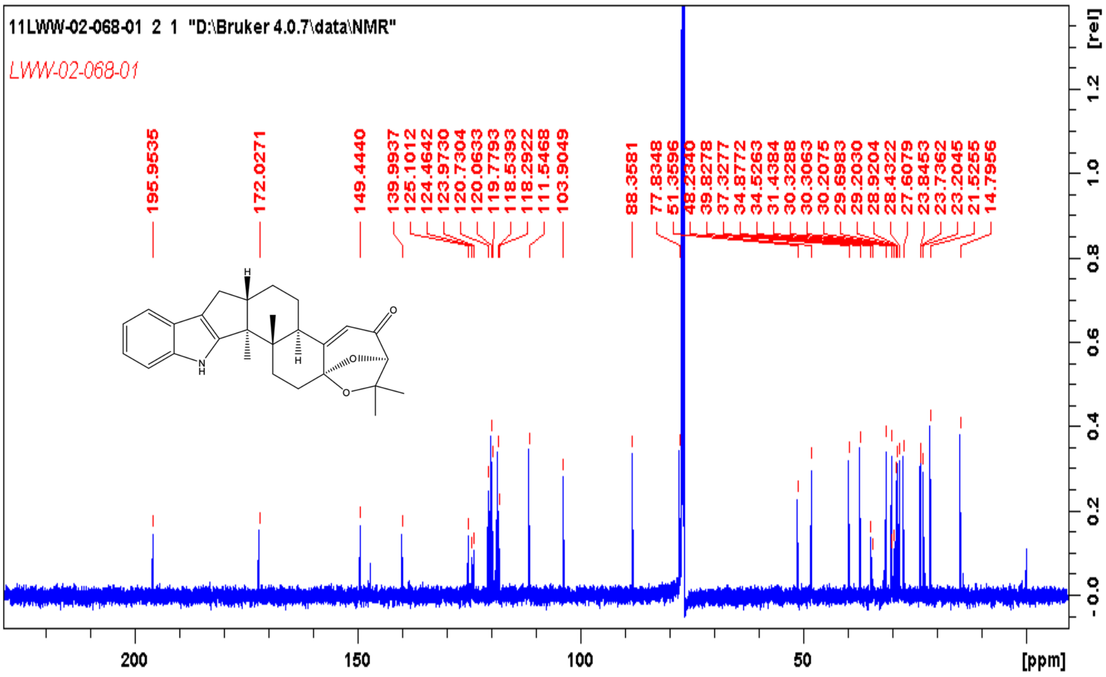

Figure S42. ^{13}C NMR (150 MHz, $CDCl_3$) spectrum of paspalicine (**11**)

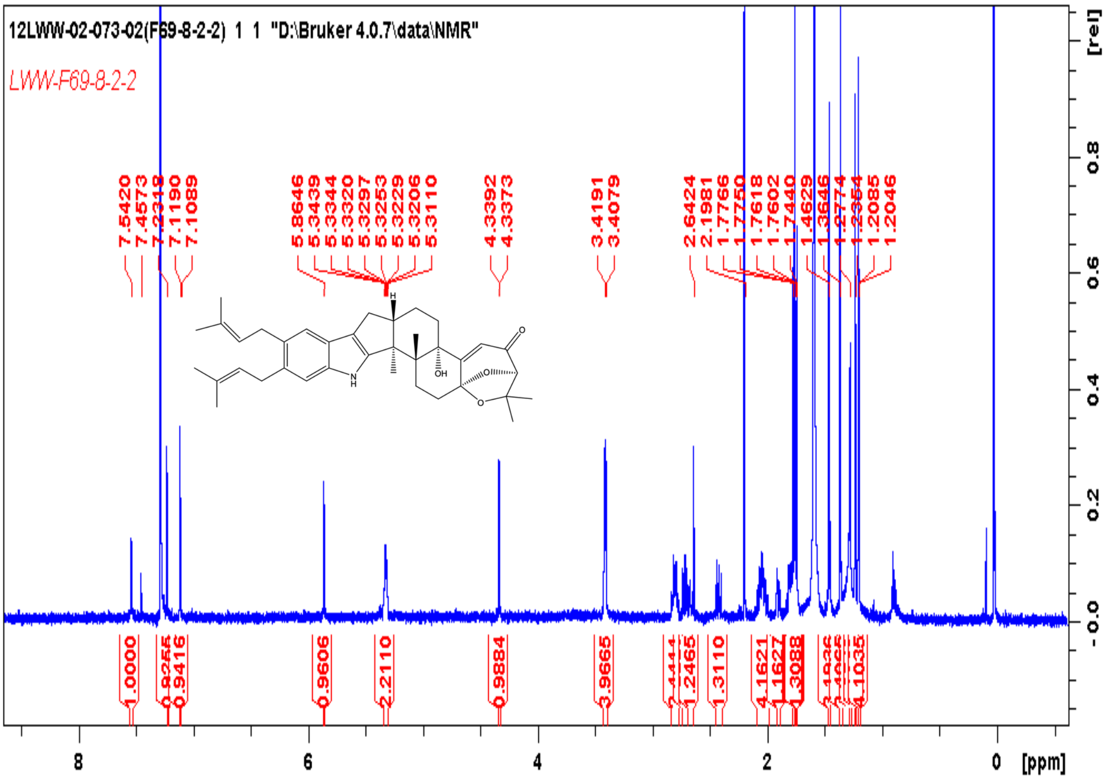

Figure S43. ^{1}H NMR (600 MHz, $CDCl_3$) spectrum of sherinine K (**12**)

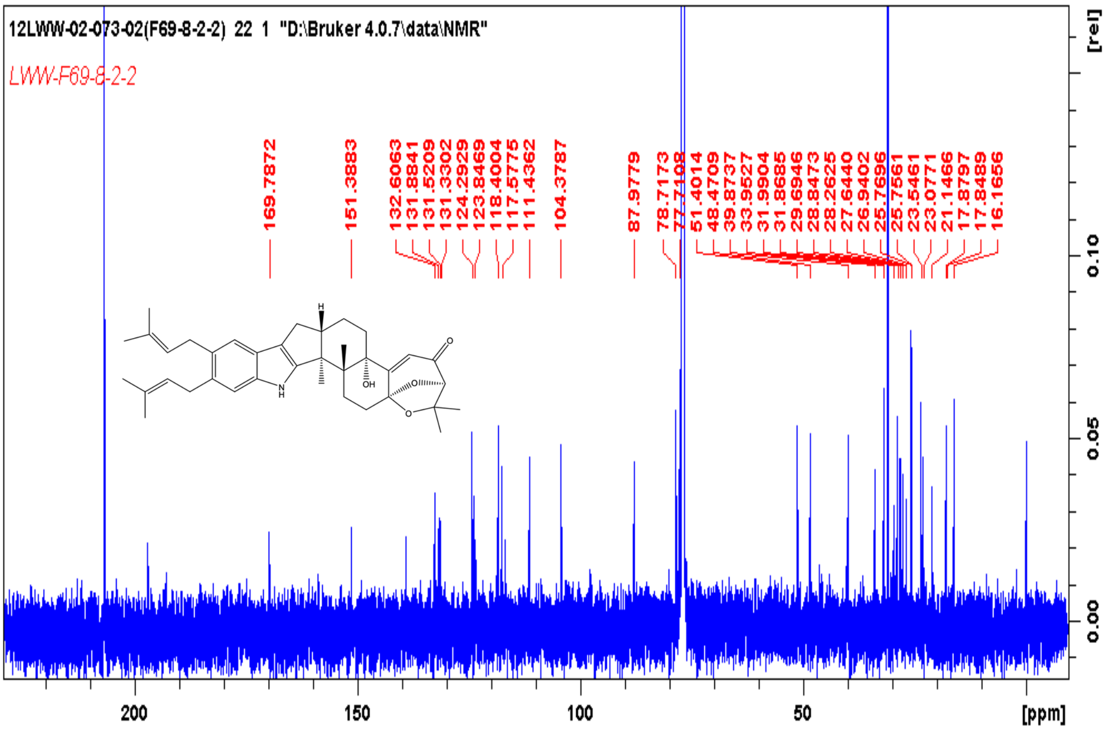

Figure S44. ^{13}C NMR (150 MHz, $CDCl_3$) spectrum of sherinine K (**12**)

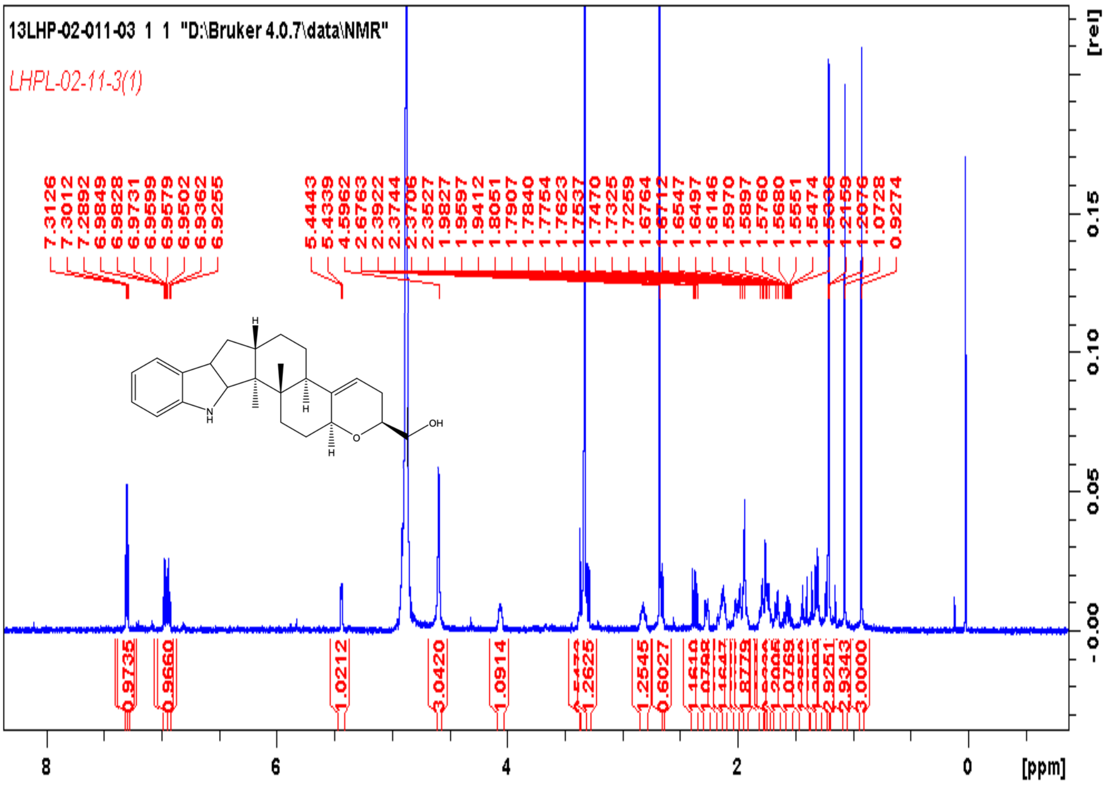

Figure S45. ^{1}HNMR (600 MHz, $CDCl_3$) spectrum of 3-deoxo-4b-deoxy paxillin (**13**)

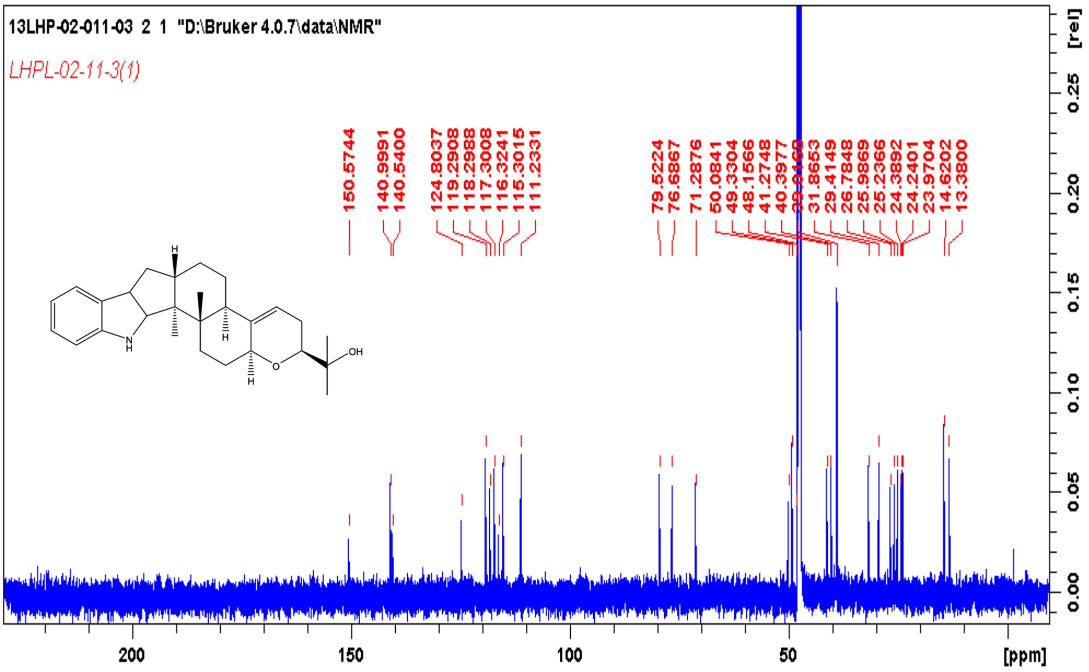

Figure S46. ^{13}C NMR (150 MHz, $CDCl_3$) spectrum of 3-deoxo- 4b-deoxy paxilline (**13**)

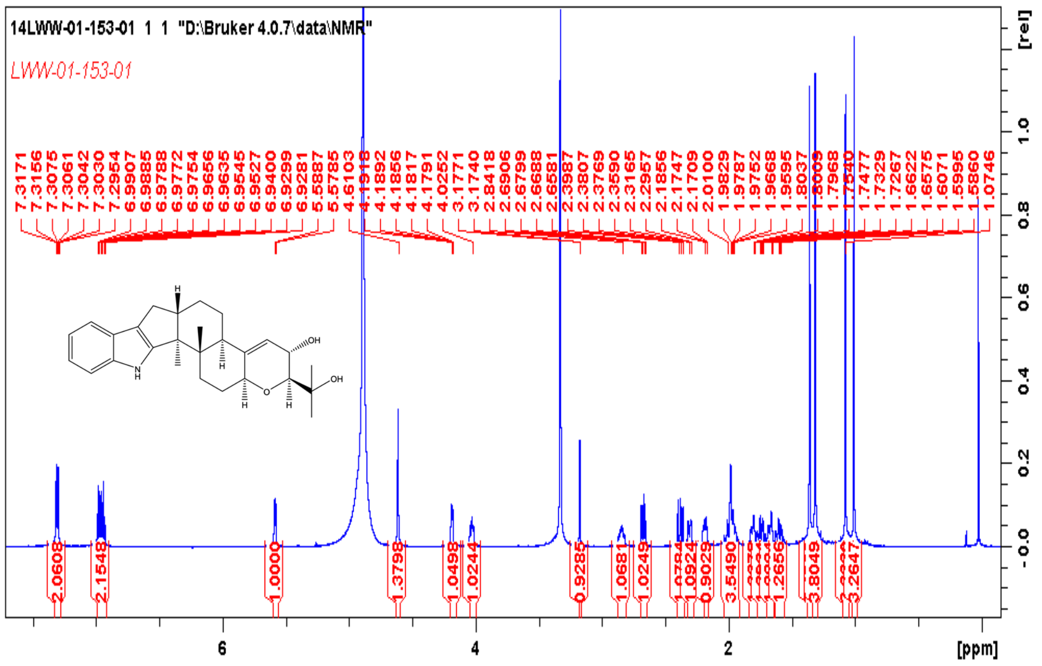

Figure S47. ^{1}H NMR (600 MHz, $CDCl_3$) spectrum of 10-hydroxy-13-desoxy paxilline (**14**)

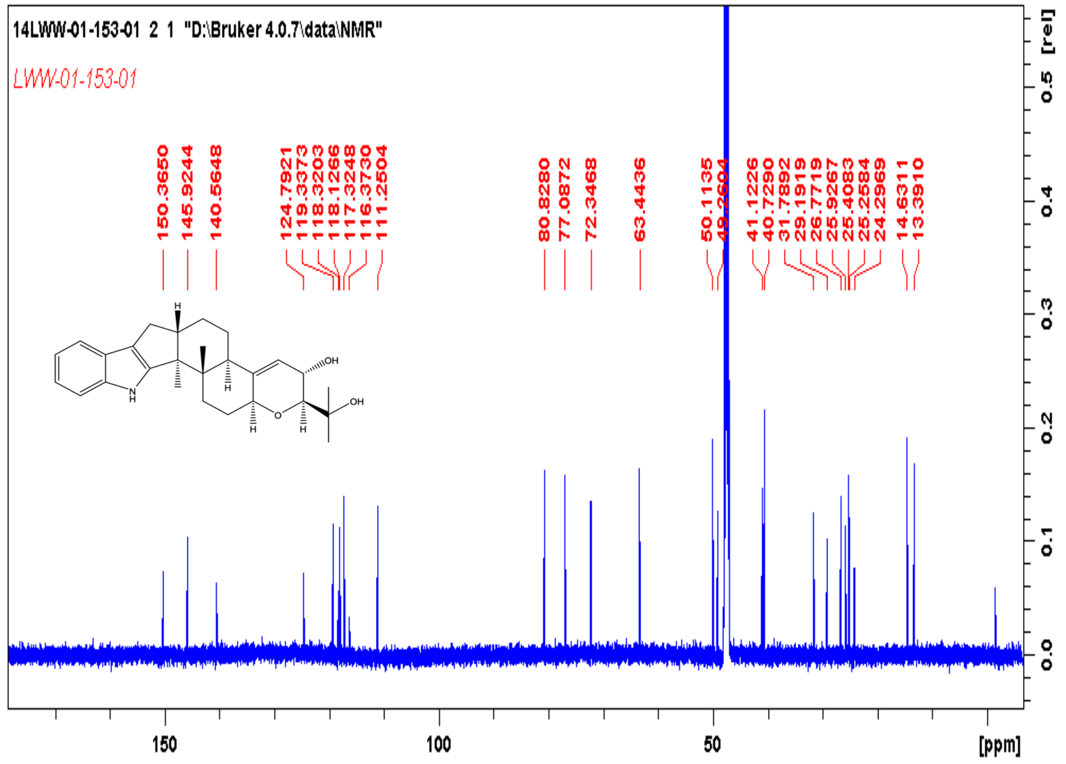

Figure S48. ^{13}C NMR (150 MHz, $CDCl_3$) spectrum of 10-hydroxy-13-desoxy paxilline (**14**)

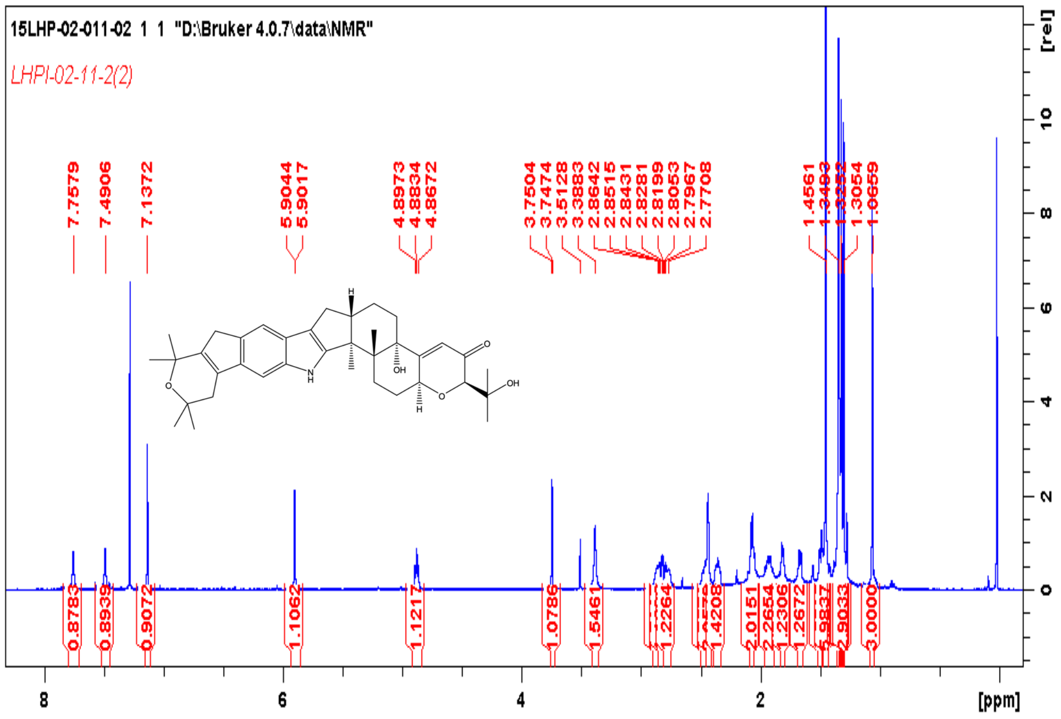

Figure S49. ^{1}H NMR (600 MHz, $CDCl_3$) spectrum of pyrapaxilline (1**5**)

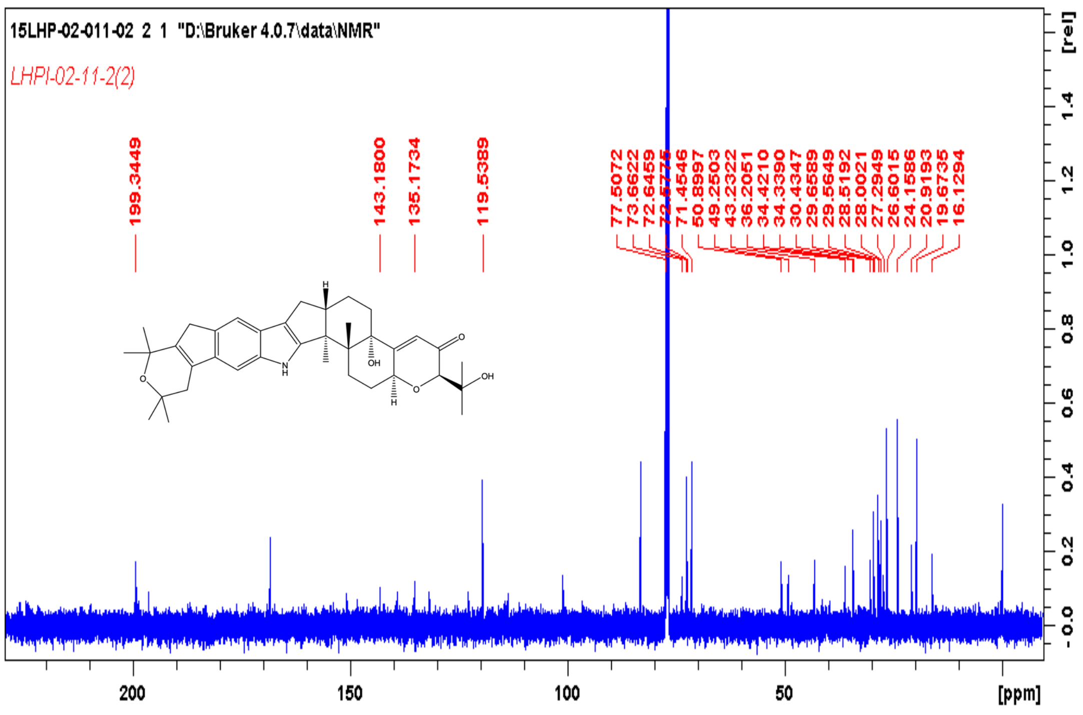

Figure S50. ^{13}C NMR (150 MHz, $CDCl_3$) spectrum of pyrapaxilline (**15**)

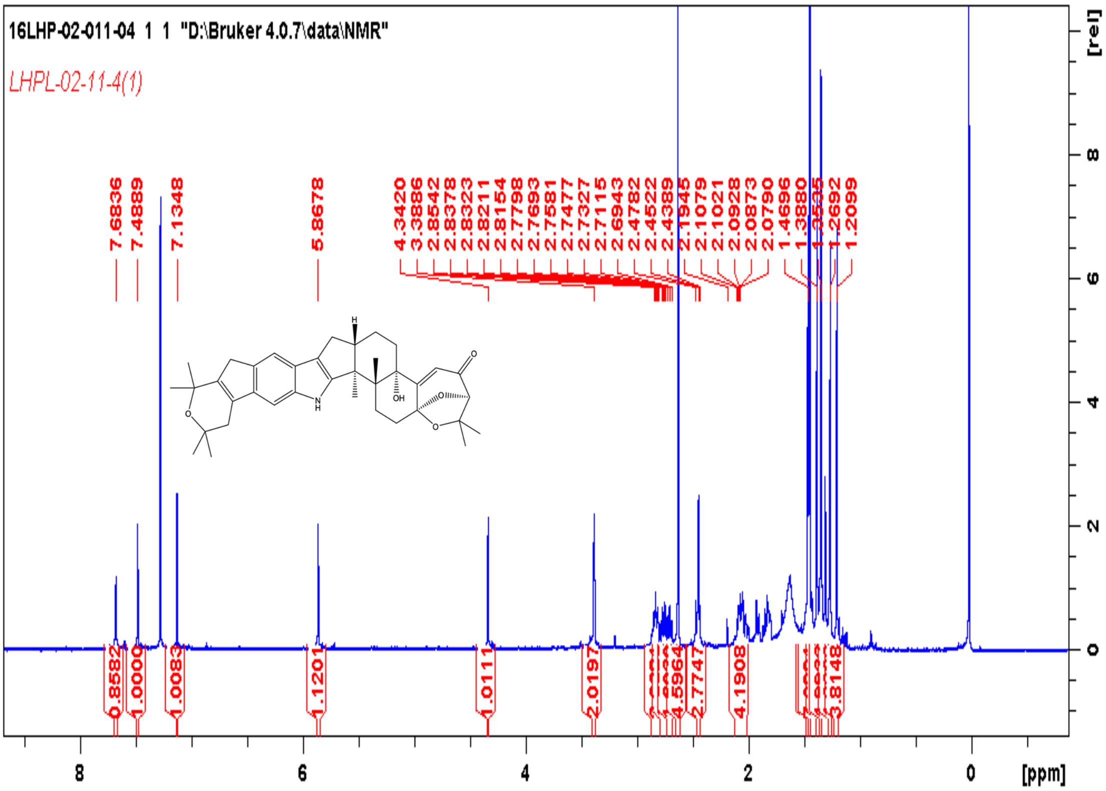

Figure S51. ^{1}H NMR (600 MHz, $CDCl_3$) spectrum of shearinine (**16**)

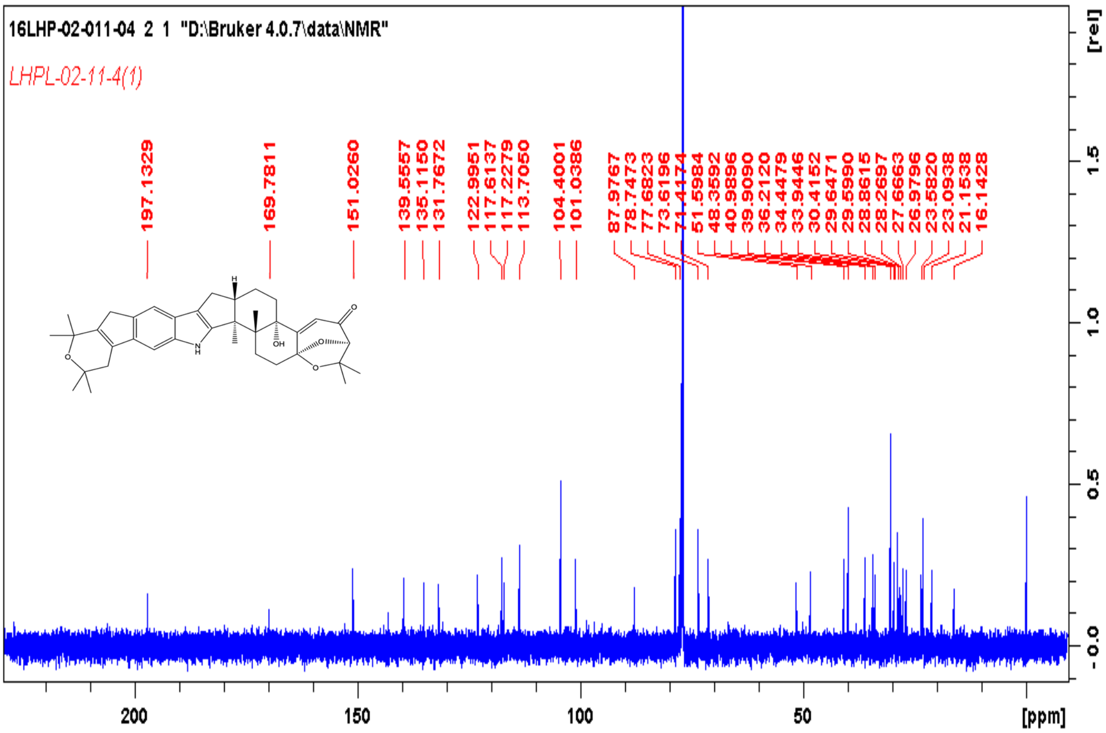

Figure S52. ^{13}C NMR (150 MHz, $CDCl_3$) spectrum of shearinine (**16**)

National Center for Organic Mass Spectrometry in Shanghai
Shanghai Institute of Organic Chemistry
Chinese Academic of Sciences
High Resolution MS ESI REPORT

Instrument: Thermo Scientific Q Exactive HF Orbitrap-FTMS

Card Serial Number: H-W190566

Sample Serial Number: LWW-02-049-03

Operator: QHL Date: 2019/06/25

Operation Mode: ESI Positive Ion Mode

Elemental composition search on mass 480.35

m/z= 475.35-485.35

m/z	Theo. Mass	Delta (ppm)	RDB equiv.	Composition
480.3474	480.3472	0.33	9.5	$C_{31}H_{46}O_3N$
	480.3479	-1.11	0.0	$C_{25}H_{52}O_6S$
	480.3466	1.69	0.5	$C_{23}H_{50}O_5N_3S$
	480.3459	3.13	10.0	$C_{29}H_{44}O_2N_4$
	480.3492	-3.89	5.0	$C_{26}H_{48}O_2N_4S$

Figure S53. HR-ESI-MS data report of paspaline C (**1**)

National Center for Organic Mass Spectrometry in Shanghai
Shanghai Institute of Organic Chemistry
Chinese Academic of Sciences
High Resolution MS ESI REPORT

Instrument: Thermo Scientific Q Exactive HF Orbitrap-FTMS

Card Serial Number: H-W190558

Sample Serial Number: LWW-01-139-01

Operator: QHL Date: 2019/06/25

Operation Mode: ESI Positive Ion Mode

Elemental composition search on mass 504.31

m/z= 499.31-509.31

m/z	Theo. Mass	Delta (ppm)	RDB equiv.	Composition
504.3087	504.3084	0.58	9.5	$C_{30}H_{43}O_4NNa$
	504.3082	1.12	8.0	$C_{29}H_{44}O_7$
	504.3108	-4.19	12.5	$C_{32}H_{42}O_4N$

Figure S54. HR-ESI-MS data report of paspaline D (**2**)

National Center for Organic Mass Spectrometry in Shanghai
Shanghai Institute of Organic Chemistry
Chinese Academic of Sciences
High Resolution MS ESI REPORT

Instrument: Thermo Scientific Q Exactive HF Orbitrap-FTMS

Card Serial Number: H-W190564

Sample Serial Number: LWW-02-048-03

Operator: QHL Date: 2019/06/25

Operation Mode: ESI Positive Ion Mode

Elemental composition search on mass 546.32

m/z= 541.32-551.32

m/z	Theo. Mass	Delta (ppm)	RDB equiv.	Composition
546.3217	546.3214	0.48	13.5	$C_{34}H_{44}O_5N$
	546.3227	-1.97	18.5	$C_{35}H_{40}ON_5$
	546.3201	2.93	14.0	$C_{32}H_{42}O_4N_4$

Figure S55. HR-ESI-MS data report of paxilline B (**3**)

National Center for Organic Mass Spectrometry in Shanghai
Shanghai Institute of Organic Chemistry
Chinese Academic of Sciences
High Resolution MS ESI REPORT

Instrument: Thermo Scientific Q Exactive HF Orbitrap-FTMS

Card Serial Number: H-W190562

Sample Serial Number: LWW-02-048-02

Operator: QHL Date: 2019/06/25

Operation Mode: ESI Positive Ion Mode

Elemental composition search on mass 546.32

m/z= 541.32-551.32

m/z	Theo. Mass	Delta (ppm)	RDB equiv.	Composition
546.3220	546.3214	1.14	13.5	$C_{34}H_{44}O_5N$
	546.3227	-1.31	18.5	$C_{35}H_{40}ON_5$
	546.3201	3.59	14.0	$C_{32}H_{42}O_4N_4$

Figure S56. HR-ESI-MS data report of paxilline C (**4**)

National Center for Organic Mass Spectrometry in Shanghai
Shanghai Institute of Organic Chemistry
Chinese Academic of Sciences
High Resolution MS ESI REPORT

Instrument: Thermo Scientific Q Exactive HF Orbitrap-FTMS

Card Serial Number: H-W190560

Sample Serial Number: LWW-02-003-06

Operator: QHL Date: 2019/06/25

Operation Mode: ESI Positive Ion Mode

Elemental composition search on mass 450.26

m/z= 445.26-455.26

m/z	Theo. Mass	Delta (ppm)	RDB equiv.	Composition
450.2638	450.2639	-0.21	11.5	$C_{28} H_{36} O_4 N$
	450.2642	-0.82	13.0	$C_{29} H_{35} O N_2 Na$
	450.2628	2.16	13.5	$C_{27} H_{33} N_5 Na$
	450.2625	2.77	12.0	$C_{26} H_{34} O_3 N_4$
	450.2652	-3.18	16.5	$C_{29} H_{32} N_5$
	450.2660	-4.93	0.0	$C_{17} H_{39} O_8 N_4 Na$

Figure S57. HR-ESI-MS data report of paxilline D (**5**)

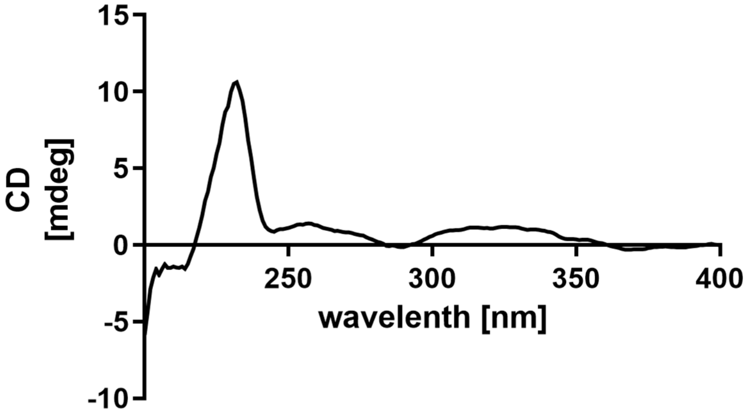

Figure S58. ECD spectrum of paspaline C (**1**)

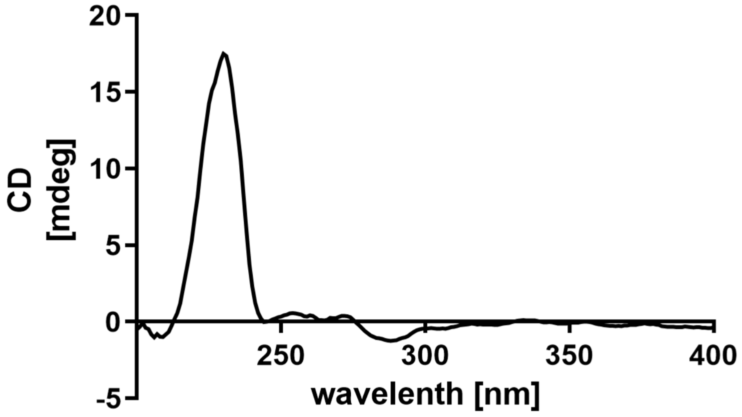

Figure S59. ECD spectrum of paspaline D (**2**)

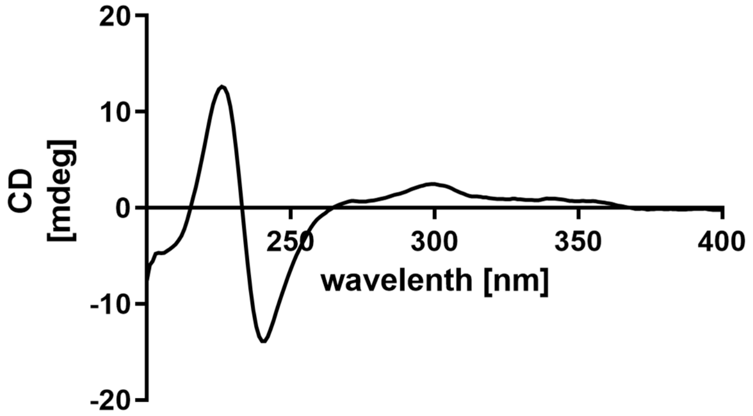

Figure S60. ECD spectrum of paxilline B (**3**)

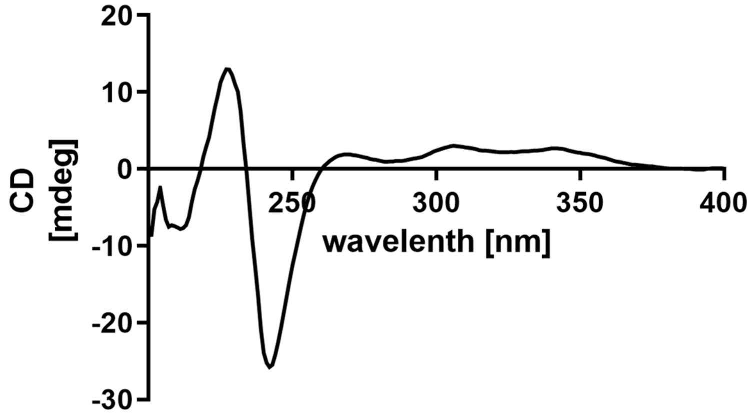

Figure S61. ECD spectrum of paxilline C (**4**)

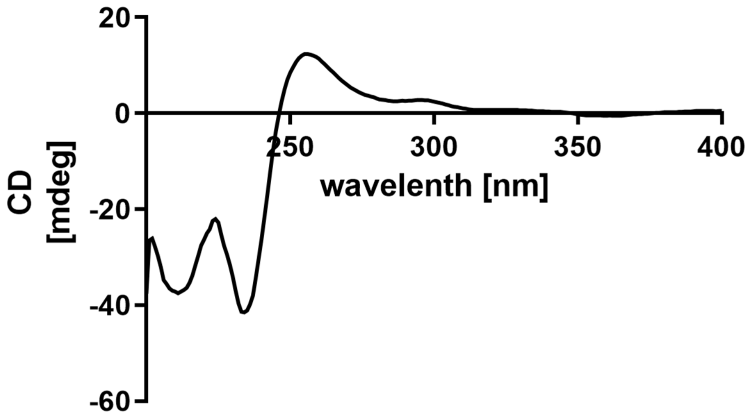

Figure S62. ECD spectrum of paxilline D (**5**)

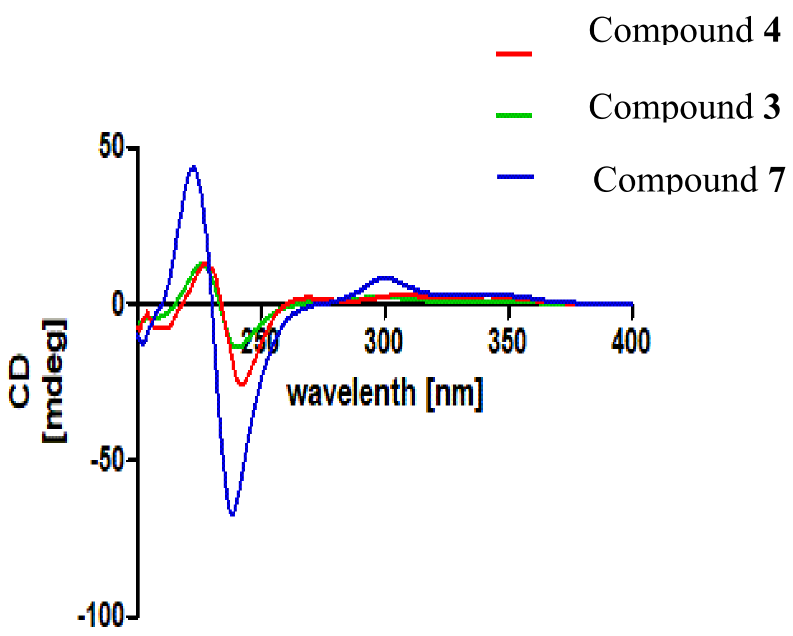

Figure S63. ECD spectrum of compound **3**, **4** and **7**

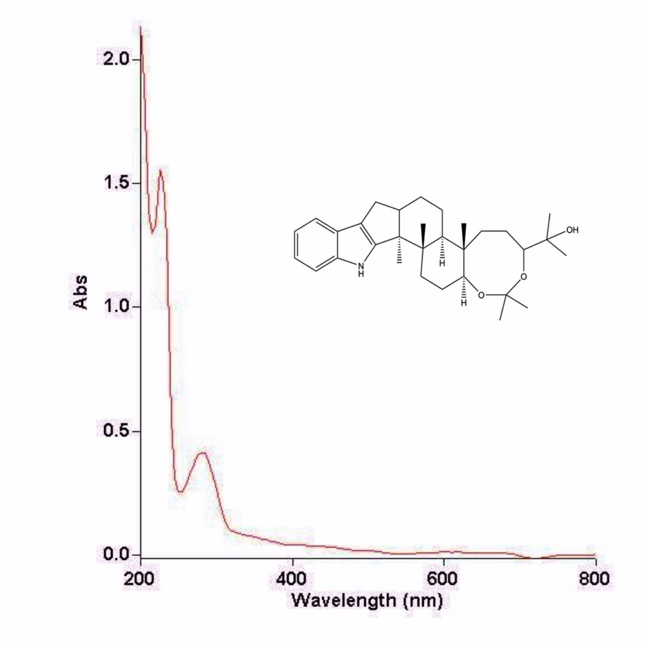

Figure S64. UV spectrum of paspaline C (**1**)

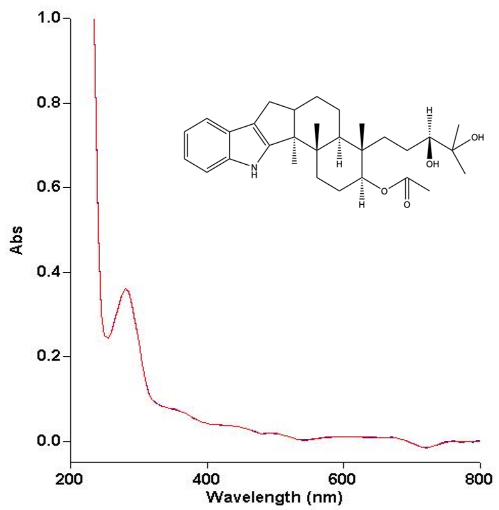

Figure S65. UV spectrum of paspaline D (**2**)

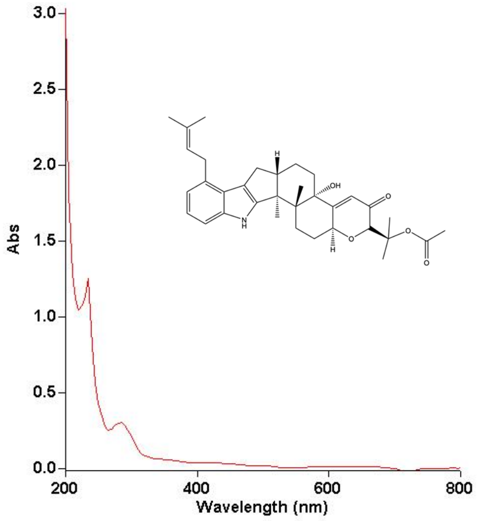

Figure S66. UV spectrum of paxilline B (**3**)

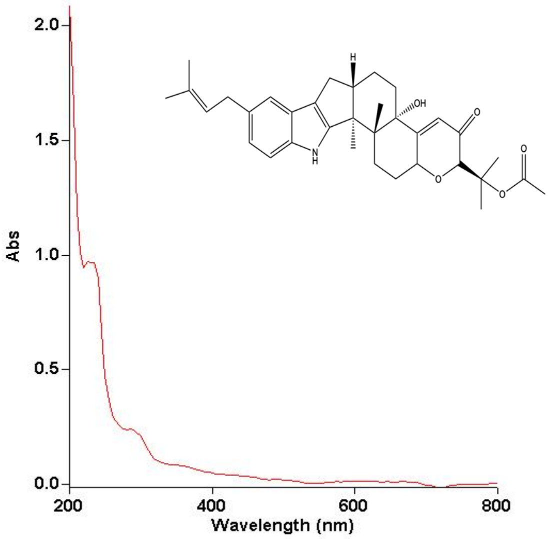

Figure S67. UV spectrum of paxilline C (**4**)

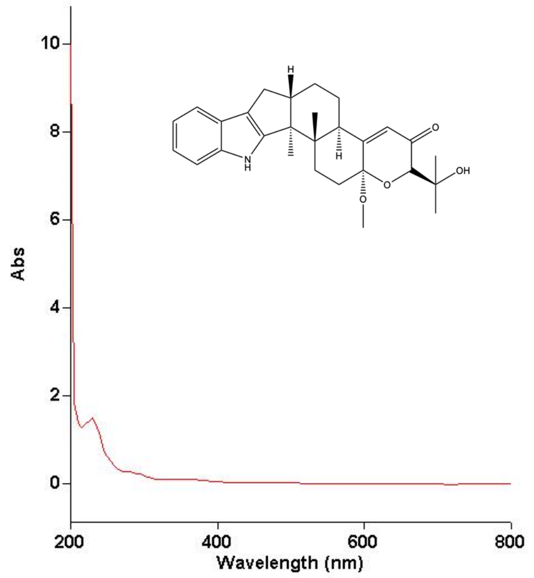

Figure S68. UV spectrum of paxilline D (**5**)

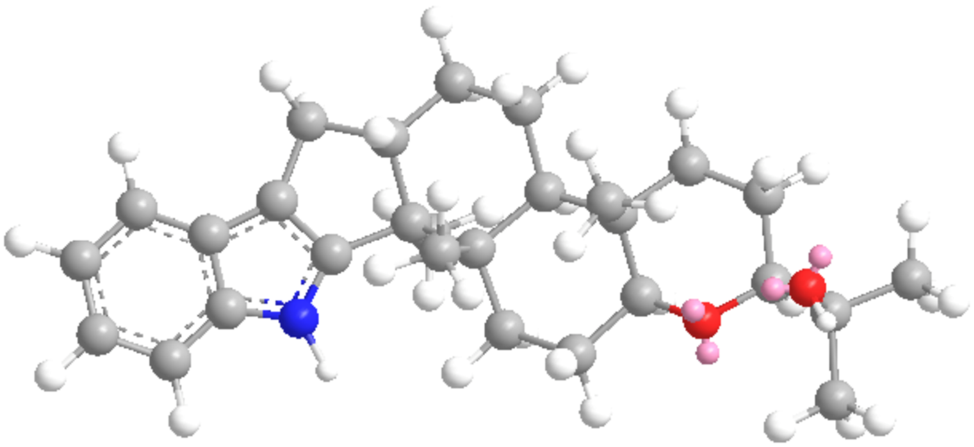

Figure S69. X-ray single crystal diffraction strucutre of paspaline (**6**)
